# Exploring Tau Fibril-Disaggregating and Antioxidating Molecules Binding to Membrane-Bound Amyloid Oligomers Using Machine Learning-Enhanced Docking and Molecular Dynamics

**DOI:** 10.3390/molecules29122818

**Published:** 2024-06-13

**Authors:** Luthary Segura, Natalia Santos, Rafael Flores, Donald Sikazwe, Miles McGibbon, Vincent Blay, Kwan H. Cheng

**Affiliations:** 1 Neuroscience Department, Trinity University, San Antonio, TX 78212, USA; lsegura1@trinity.edu; 2 Physics Department, Trinity University, San Antonio, TX 78212, USA; nsantos@trinity.edu; 3 Pharmaceutical Sciences Department, Feik School of Pharmacy, University of the Incarnate Word, San Antonio, TX 78209, USA; raflore7@uiwtx.edu (R.F.); sikazwe@uiwtx.edu (D.S.); 4 Institute of Quantitative Biology, Biochemistry and Biotechnology, University of Edinburgh, Edinburgh EH9 3BF, UK; milesmcgibbon@gmx.us; 5 Department of Microbiology and Environmental Toxicology, University of California at Santa Cruz, Santa Cruz, CA 95064, USA; vroger@ucsc.edu

**Keywords:** neurodegenerative disease, molecular drug docking, machine learning, amyloid oligomers, drug–protein interactions, protein folding, drug design, cheminformatics

## Abstract

Intracellular tau fibrils are sources of neurotoxicity and oxidative stress in Alzheimer’s. Current drug discovery efforts have focused on molecules with tau fibril disaggregation and antioxidation functions. However, recent studies suggest that membrane-bound tau-containing oligomers (mTCOs), smaller and less ordered than tau fibrils, are neurotoxic in the early stage of Alzheimer’s. Whether tau fibril-targeting molecules are effective against mTCOs is unknown. The binding of epigallocatechin-3-gallate (EGCG), CNS-11, and BHT-CNS-11 to in silico mTCOs and experimental tau fibrils was investigated using machine learning-enhanced docking and molecular dynamics simulations. EGCG and CNS-11 have tau fibril disaggregation functions, while the proposed BHT-CNS-11 has potential tau fibril disaggregation and antioxidation functions like EGCG. Our results suggest that the three molecules studied may also bind to mTCOs. The predicted binding probability of EGCG to mTCOs increases with the protein aggregate size. In contrast, the predicted probability of CNS-11 and BHT-CNS-11 binding to the dimeric mTCOs is higher than binding to the tetrameric mTCOs for the homo tau but not for the hetero tau–amylin oligomers. Our results also support the idea that anionic lipids may promote the binding of molecules to mTCOs. We conclude that tau fibril-disaggregating and antioxidating molecules may bind to mTCOs, and that mTCOs may also be useful targets for Alzheimer’s drug design.

## 1. Introduction

Alzheimer’s disease (AD) is a prevalent and devastating condition, with nearly 35 million people affected globally [1]. Despite the increase in cases of AD in recent years, its complex mechanisms remain unclear, and no effective treatments to slow or reverse its progression have been found. A few current treatments include Lecanemab, aducanumab, donepezil, galantamine, rivastigmine, and memantine [2,3]. Of these, Lecanemab and aducanumab are the only ones used to clear Aβ plaques, while the remaining four focus on treating symptoms. In addition to the high cost, costly requirement of regular magnetic resonance imaging monitoring, and the restricted insurance coverage for Lecanemab and aducanumab, these treatment methods have resulted in side effects like amyloid-related imaging abnormalities in edema and hemorrhage [4,5]. This news has pushed the community to look for other targets, paving the way for tau-targeting therapies for AD. However, most strategies currently undergoing clinical trials have focused on the N-terminal of extracellular tau [6]. Non-monoclonal antibody treatment, e.g., using a synthesized peptide to chelate excessive iron ions in order to reduce free radicals in the brain, has also been proposed [7]. Here, we employ computational methods to explore tau targets from both intracellular and extracellular regions and the binding specificity of tau fibril-disaggregating small molecules. Unlike FDA-approved small molecule drugs like donepezil, galantamine, rivastigmine, and memantine, the compounds proposed in this study have been selected and designed with tau protein structures as the focus target.

The intracellular neurofibril entanglements of tau proteins in brain neurons are well-known histological markers of AD. These highly ordered tau fibrils are cytotoxic and sources of oxidative stress in AD development [8,9,10,11,12,13,14]. However, recent in vivo and in vitro studies [15,16,17,18,19,20,21,22,23,24,25,26] strongly suggest that the smaller, less-ordered membrane-bound tau oligomers are also cytotoxic to neurons in early AD pathogenesis. This is pertinent considering that patients with AD appear symptom-free in preclinical stages, although neurotoxicity is present in the brain [1]. The normal function of this intrinsically disordered protein is to regulate microtubule stability and axonal transport [27]; however, its detachment from the microtubules results in misfolding and accumulation in the cytosol, acting as seeds that propagate between cells to induce misfolding of other tau molecules [19,20,21]. These tau oligomers with seeding characteristics have exhibited disruption of proteostatic mechanisms at synapses, oxidative stress through damage to the mitochondria, and synaptic loss in AD patients [27,28]. Recent clinical, in vitro, and in silico studies [9,29,30,31,32,33,34] have further indicated that tau can interact or cross-seed with amylin, an amyloid protein associated with type 2 diabetes, to form membrane-bound hetero tau–amylin oligomers. These heterooligomers contribute to the molecular crosstalk between AD and type 2 diabetes [30,34]. Notably, a higher incidence of AD is found in patients with type 2 diabetes compared to individuals without comorbidities: 81% of people with AD also have type 2 diabetes or abnormal fasting glucose levels [35]. To date, tau fibrils have been the primary therapeutic target in AD, and virtual drug discovery studies have mostly focused on developing small molecules for tau fibril disaggregation and elimination of reactive oxygen species or antioxidation [6,15,24]. Therapeutic interventions targeting membrane-bound tau-containing oligomers or mTCOs, including homo tau and hetero tau–amylin aggregates at the early stage of AD, could offer new possibilities [36]. The core objective of this study is to determine if in silico mTCOs are feasible targets for therapeutic interventions in AD by screening the binding of relevant compounds.

Advances in Cryo-EM technology have yielded atomistic structures of tau fibrils in brain autopsy samples from AD patients [11,13]. These clinically relevant tau fibril structures have been used as targets for tau fibril-disaggregating molecules such as epigallocatechin-3-gallate (EGCG) and CNS-11 [13]. EGCG is a natural molecule with tau fibril disaggregation and antioxidation functions that is extracted from green tea [13,37]. However, EGCG has poor drug-like properties [13]. On the other hand, CNS-11, with a structure resembling that of EGCG, is a drug-like molecule with a similar tau fibril disaggregation function [13]. However, CNS-11 does not have an antioxidation function like EGCG. This study introduces a drug-like molecule, BHT-CNS-11, with potential tau fibril disaggregation and antioxidation functions. BHT-CNS-11 is a hybrid of CNS-11 and butylated hydroxytoluene (BHT), a known antioxidant [38]. Whether the above tau fibril-disaggregating molecules with or without antioxidation functions can target the toxic mTCOs during the early stages of AD progression remains unknown and is the focus of this computational study. Due to the nature of mTCO protein folding in obtaining more protein pockets, we expect that the tau fibril-targeting molecules will bind better with mTCOs compared with tau fibrils.

Unlike tau fibrils, experimental atomistic structures of toxic mTCOs are still not available. Therefore, in silico and physiologically relevant mTCO structures are needed. Using physics-based multiscale molecular dynamics (MD) simulations, we have successfully designed and simulated stable mTCOs of sizes up to tetramers on various model neuronal raft membranes under physiologically relevant conditions [18,32,39,40]. The raft membranes correspond to the cytoplasmic and exoplasmic leaflets of the neuronal plasma membrane containing anionic phosphatidylserine (PS) and monosialotetrahexosylganglioside (GM1) lipids, or PS-raft and GM-raft, respectively. In addition, membranes without anionic lipids (CO-raft) were also investigated as controls. These mTCOs with different protein contents, aggregation sizes, and various raft membranes represent our in silico targets in this work.

Using AutoDock Vina, a molecular docking program [41], and a recent machine-learning (ML) docking-pose-rescoring algorithm [42], SCORCH (Scoring Consensus for RMSD-based Classification of Hits), we performed docking and rescoring of EGCG, CNS-11, and BHT-CNS-11 to our in silico mTCOs, as well as to experimental tau fibril structures as controls. Here, SCORCH, trained on datasets containing experimental protein-compound complex structures and docking decoys, provides improved pose identification of compound–oligomers and compound–fibril complexes.

The results suggest potentially stronger binding of the small molecules to mTCOs than to tau fibrils, with EGCG progressively increasing affinity to mTCOs with protein aggregation size. CNS-11 and BHT-CNS-11 are shown to bind more tightly to the dimeric homo tau aggregates than to the tetrameric ones, but the reverse is true for the hetero tau–amylin aggregates. Additionally, results suggest that the anionic lipids in the raft membrane could strengthen the binding of the small molecules to the mTCOs. Our results address critical pathophysiological pathways of tauopathies involving early tau-containing oligomers and tau fibrils in the intracellular and extracellular regions of neuronal plasma membranes linked to AD. The combination of physics- and ML-based computational tools provides new insights into the druggability of toxic mTCOs and could prove useful for the future design of novel therapeutics against AD.

## 2. Results

### 2.1. Multipurpose Small Molecules and Targets

To develop multipurpose drugs with specific functions for therapeutic interventions against AD, small molecules with known or predicted enzyme inhibition, antioxidant, and tau fibril-disaggregation activities were investigated. Eight compounds, 6QH, donepezil (E20), BHT, EGCG, CNS-11, BHT-6QH, BHT-E20, and BHT-CNS-11, were examined, and their structure–activity relationships are summarized in Table 1. 6QH [43] and E20 [44] are known inhibitors of the 3β isoform of glycogen synthase kinase (GSK-3β) and acetylcholinesterase (AChE) and are associated with the hyperphosphorylation of tau and the degradation of acetylcholine in neurons, respectively. Inhibitions of GSK-3β and AChE can disrupt the tau aggregation pathway [45,46,47] and improve acetylcholine signaling [44]. Moreover, the phosphorylation of tau by GSK-3β regulates the expression of AChE, provoking an imbalance in cholinergic activity and a decrease in acetylcholine in the cell [48]. The natural compounds, BHT from algae and EGCG from green tea, are well-known antioxidants [37,38]. Both EGCG and CNS-11 were found to have tau fibril disaggregation activities in brain autopsy samples from AD patients [13]. Note that 6QH, E20, and CNS-11 do not have antioxidant activity. To add antioxidation activity to these molecules, a BHT moiety was covalently attached to the pharmacophore regions of these molecules to create new BHT-containing hybrids, BHT–6QH [43], BHT–E20 [49], and BHT–CNS-11. The first two hybrids have demonstrated dual enzyme inhibition and antioxidant activities, while the last hybrid has potential antioxidant and tau fibril disaggregation activities that await experimental validation.

The target structures in this study are summarized in Table 2. We used X-ray crystal structures of GSK-3β [46] and AChE [44] as our enzyme targets. For the amyloid targets, both experimental fibrillar and in silico oligomeric structures were used.

The amyloid fibril structures studied include tau-PHF-A, tau-PHF-B, and tau-PHF-C structures, all derived from Cryo-EM images of tau fibrils from brain samples with AD. Tau-PHF-A and tau-PHF-B refer to the fibrils in the absence and presence of EGCG embedded at the interface of two symmetric tau-pentamers, provided by Seidler et al. [13]. On the other hand, tau-PHF-C refers to another Cryo-EM structure without any compounds, provided by Fitzpatrick et al. [11]. We also included amylin fibrils derived from Cryo-EM images provided by Cao et al. [50], which served as controls. For more details, see Materials and Methods.

The amyloid oligomers modeled include eight types of in silico membrane-bound oligomers, each with different protein contents (tau alone, amylin alone, and tau–amylin hybrid), aggregate sizes (monomer, dimer, and tetramer), and membrane surfaces (CO-, PS-, and GM-rafts). As described in the Materials and Methods section, these in silico membrane-bound oligomers were created using multiscale MD simulations under physiologically relevant conditions. Specifically, our homo tau oligomers include monomeric (1tau), dimeric (2tau), and tetrameric (4tau) tau aggregates. Our homo amylin oligomers include monomeric (1am), dimeric (2am), and tetrameric (4am) amylin aggregates. Our hetero tau oligomers include dimeric (1tam) and tetrameric (2tam) tau–amylin hybrid aggregates. We primarily focus on membrane-bound tau-containing oligomers, or mTCOs, i.e., 1tau, 2tau, 4tau, 1tam, and 2tam. The membrane-bound amylin oligomers, i.e., 1am, 2am, and 4am, are used as controls. Overall, our in silico amyloid oligomer targets comprised 8 protein oligomer types, each on 3 different membrane surfaces, and 3 replicates, a total of 72 independently simulated oligomer-based target structures. See Materials and Methods for details.

In summary, our docking study involves 8 compounds against 78 targets: 2 enzymes (GSK-3β and AChE), 4 Cryo-EM-derived amyloid fibrils, and 72 in silico amyloid oligomers. Our primary focus was on exploring tau fibril-disaggregating and antioxidant molecules targeting tau fibrils and mTCOs.

### 2.2. Predicted Pharmacokinetic and Toxicity Profiles of Compounds

We used the open-source cheminformatic tools, pkCSM [51] and ADMET Lab 3.0 [52], to predict the chemical absorption, distribution, metabolism, excretion, and toxicity (ADMET) profiles of the compounds. In addition, we used the online MolSoft tool (https://www.molsoft.com/servers.html (accessed on 8 June 2024)) to predict the molecular properties and drug-likeness of the compounds.

The absorption properties of the compounds were evaluated based on the calculated intestinal absorbance and water solubility values, and the results are shown in Figure S1. All 8 compounds have >60% predicted intestinal absorbance, optimal for drug-like compounds. For the water solubility properties given in Figure S1B, EGCG, BHT-CNS-11, and 6QH have optimal drug-like water solubility (Log S) values > −4, while the other compounds have Log S values between −4 and −6, within the range of moderately soluble drug-like compounds. As shown in Figure S1C, CNS-11, BHT, 6QH, and E20 have lipophilicity (log P) values within the range of 2–5, indicating that these compounds may have good intestinal absorption and CNS permeability, while BHT-CNS-11, BHT-6QH, and BHT-E20 exceed the upper threshold and EGCG subceeds the lower threshold [52,53]. EGCG, 6QH, and E20 have log n-octanol/water distribution coefficients at pH = 7.4 (Log D) within the range of 1–3, indicating that these compounds may reach a good balance between lipophilicity and hydrophilicity to dissolve in the body fluid and penetrate the biomembrane effectively, while the rest of the compounds exceed the upper threshold as shown in Figure S1C [52]. Note that log P corresponds to the compound’s lipophilicity without considering functional group ionization. On the other hand, log D explicitly deals with the degree of ionization and is pH-dependent. A recently proposed lipophilic metric, Fraction Lipophilicity Index (FLI), defined as 2log P − |log D|, was employed to evaluate the lipophilicity values of ionizable compounds [54]. Ionizable compounds that fall into the FLI range of 0–8 cover more than 90% of highly to moderately absorbed compounds for drug use. As shown in Figure S1C, all eight compounds except BHT-CNS-11 and BHT-6QH have less than the FLI threshold of eight. Note that the FLI value for BHT-CNS-11 slightly exceeds the threshold.

The distribution properties of the compounds were evaluated based on the calculated human steady-state volume of distribution (VDss), blood–brain barrier permeability, and cranial nervous system permeability values, and the results are shown in Figure S2. All compounds, except EGCG, have unfavorable predicted VDss values as given in Figure S2A. Note that VDss is considered too low if it is <0.15 and too high if it is >0.45. The higher the VDss, the more the compound is distributed in tissue rather than plasma. However, a low VDss indicates that a lower dose of the compound is required to achieve a given plasma concentration, while a high VDss indicates that a higher dose of the compound is needed [55]. Poor blood–brain barrier (BBB) permeability (Log BB) was observed in EGCG, BHT-6QH, and 6QH, as shown in Figure S2B. The optimal value of log BB for a compound to readily cross the blood–brain barrier is >0.3, and compounds with a Log BB < −1 are poorly distributed across the BBB. Poor cranial nervous system permeability (Log PS) was evident for EGCG and 6QH. Note that an optimal log PS value for a drug-like compound is >−0.3, while a compound with a log PS value < −1.0 is poorly distributed to the brain.

We predicted the ability of the compounds to inhibit seven enzymes with key roles in the metabolism of drugs and xenobiotics, as shown in Table S2. Most compounds were predicted to inhibit 2–3 enzymes, while CNS-11 and BHT-CNS-11 inhibited 4 enzymes.

The excretion and toxicity of the compounds were evaluated based on the predicted total clearance rate, maximum tolerated dose, and toxicity profile, and the results are shown in Figure S3 and Table S3. For excretion, all compounds, except BHT-CNS-11 and BHT-6QH, exhibit a positive clearance rate (log ml/min/kg), as given in Figure S3A. The total clearance values do not have a set ideal range and are instead used to determine the dosing rates and bioavailability of each compound. For toxicity properties, BHT-CNS-11, BHT-E20, BHT, and E20 have low maximum tolerated dose (MTD) values, as shown in Figure S3B. Note that MTD predicts the toxic dose threshold of each compound in humans. A compound with ≤0.477 log mg/kg/day is considered to have a low MTD and a compound with >0.477 log mg/kg/day is considered to have a high MTD. Finally, a toxicity profile of compounds based on their predicted carcinogenic, cardiac, and liver effects is given in Table S3. Only BHT exhibits no effects, consistent with FDA approval, while the other compounds show 1–3 effects.

The molecular and drug-likeness properties from Molsoft, including Lipinski drug-like space, Log P, Log S, polar surface area (PSA), and drug-likeness score, are presented in Table S3. Of the eight compounds, CNS-11, BHT, 6QH, and E20 fell within optimal ranges, while BHT-CNS-11 and BHT-E20 had Log P scores slightly exceeding the upper threshold. The hydroxyl groups that are found in the EGCG, BHT-CNS-11, BHT-E20, and BHT-6QH, and the amine groups of 6QH, CNS-11, BHT-CNS-11, BHT-E20, and BHT-6QH act as ionizable functional groups and can affect the pKa values listed. Traditionally, compounds with low pKa values have good solubility and poor permeability, while compounds with high pKa values have poor solubility and good permeability. In other words, the compounds with the most ionizable properties are more soluble but less permeable, e.g., EGCG, while compounds with mostly non-ionizable compounds are less soluble but more permeable, e.g., BHT-E20.

Overall, our ADMET evaluation results indicate that the newly proposed BHT-CNS-11 has reasonable drug-like properties, like CNS-11, while EGCG has poor drug-like properties. Note that E20 and BHT are an FDA-approved drug for AD and a common food additive, respectively, and have good drug-like properties based on predictions, validating the accuracy of the cheminformatic tool used.

### 2.3. Selection of Top Molecule Leads

**Figure 1:**
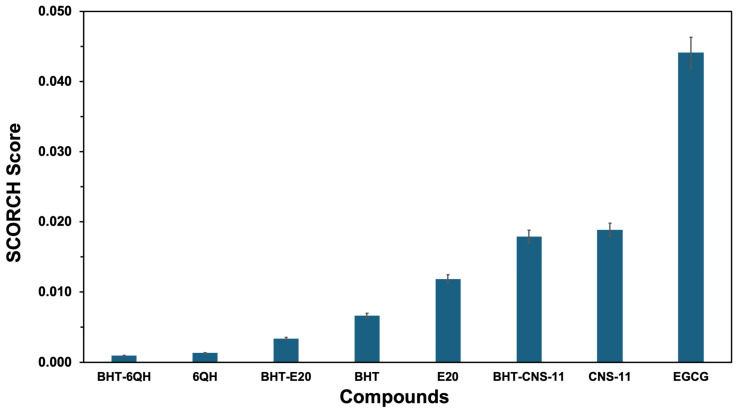
SCORCH scores for the compounds. The plot shows the average SCORCH score for different compounds across all protein structures of interest. The error bar indicates the standard deviation of the scores.

We performed extensive docking of 8 compounds to all 78 targets, including enzymes, fibrils, and amyloid oligomers. Our results are shown in Figure 1. As discussed in the Materials and Methods section, our docking workflow involves AutoDock Vina and subsequent pose rescoring using ML-based SCORCH. The mean SCORCH scores for all 8 compounds are ranked from low to high to facilitate comparison. Here, the top-ranked compounds, EGCG, CNS-11, and BHT-CNS-11, had significantly greater mean SCORCH scores than the other compounds. The compound E20, a native compound of AChE, ranked just after the top three leads. These three compounds were classified as our top molecular leads, and their scores for binding to amyloid fibrils and oligomers were systematically examined.

### 2.4. Binding to Tau Fibrils

**Figure 2:**
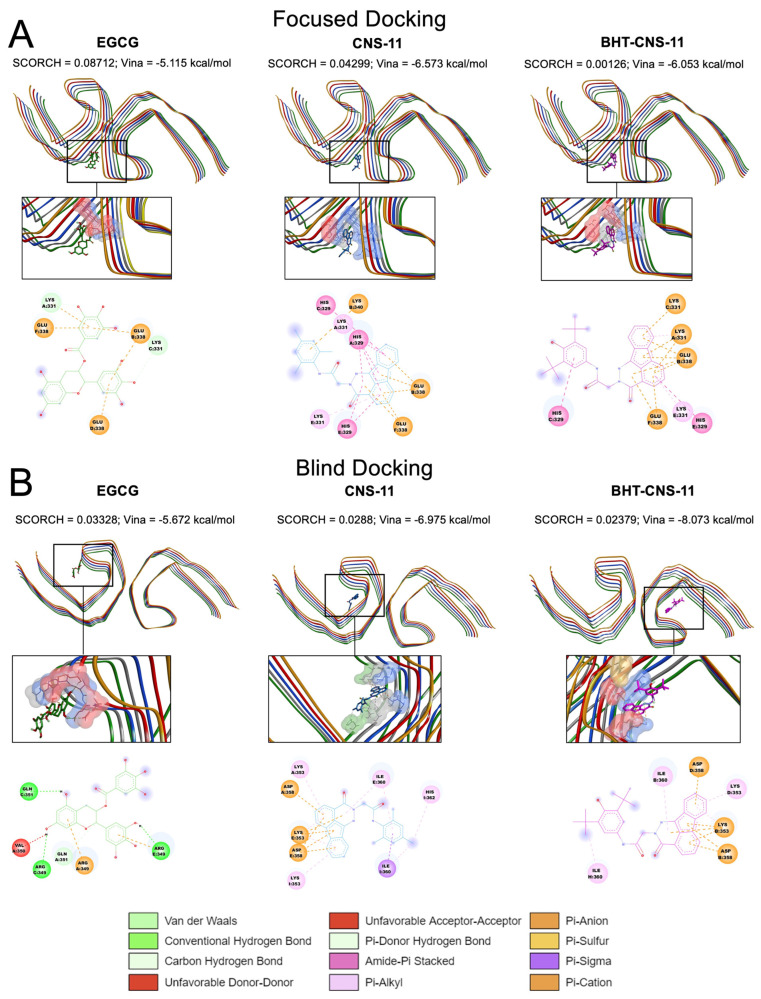
Docking of compounds to tau-PHF-B. Docking of EGCG (green), CNS-11 (blue), and BHT-CNS-11 (purple) to the target tau-PHF-B. The plots illustrate the binding location, Autodock Vina binding affinity (Vina), SCORCH score (SCORCH), and 2D chemical interactions (last rows). Focused docking of compounds to tau-PHF-B with a fixed search box of 20 Å × 16 Å × 12 Å (**A**) and blind docking (**B**) without a pre-defined search box are shown. The protein chain backbones are labeled in different colors. The protein residues that interact with the compound are labeled in licorices and surfaces in the boxes. The types of chemical interactions are color coded, as shown at the bottom of the figure.

**Figure 3:**
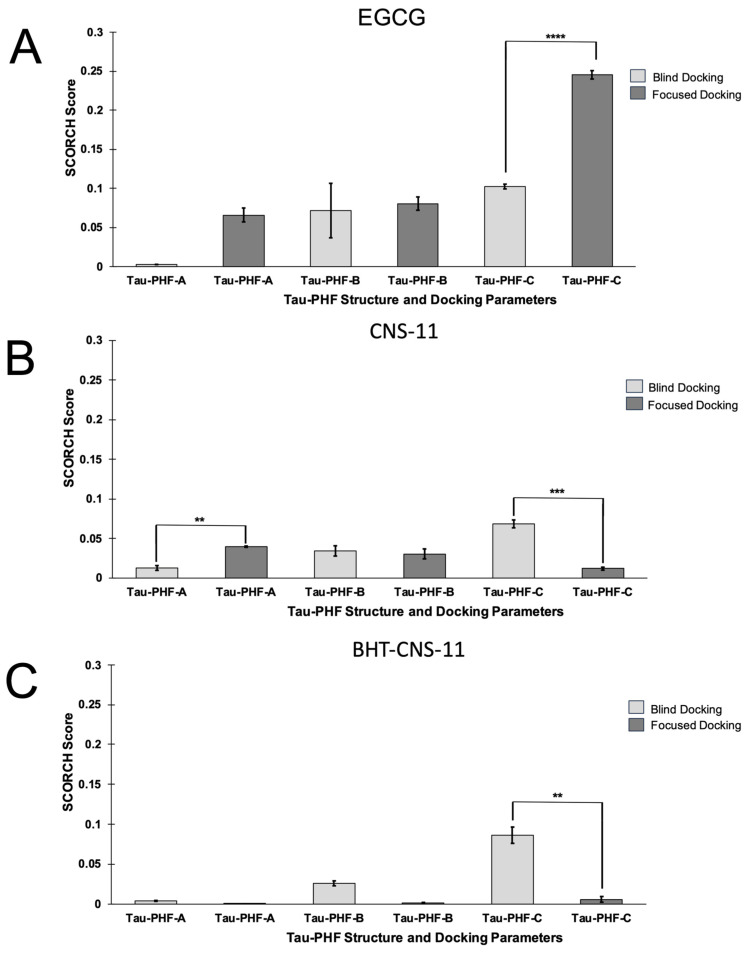
Comparison of SCORCH scores among different tau-PHF structures. Average SCORCH scores for compounds EGCG (**A**), CNS-11 (**B**), and BHT-CNS-11 (**C**) are compared across 4 iterations for each compound–target complex. Each iteration was averaged into a single SCORCH pose score and compared in independent samples using a *t*-test or Mann–Whitney U test. The scores displayed are averages of the top compound pose from each iteration. The error bar indicates the standard deviation of the mean. ** <0.01, *** <0.001, **** <0.0001.

The binding behaviors of the top three leads to all three tau fibrils, tau-PHF-A, tau-PHF-B, and tau-PHF-C, were compared. For clarity, stronger binding refers to a more negative Vina binding affinity and a higher SCORCH score. As described in Materials and Methods, tau-PHF-A and tau-PHF-B represent the Cryo-EM structures from AD brain autopsy samples, with and without EGCG bound to the cleft between two pentameric fibrils, respectively. Tau-PHF-C is a Cryo-EM structure but from different brain autopsy samples. Since EGCG, a native compound, was found in the cleft region between the two fibril pentamers of Tau-PHF-B [13] from Cryo-EM, a focused docking with a search box of 20 × 16 × 12 Å^3^ at the reported EGCG binding site was performed. Figure 2A shows the top poses identified by SCORCH for all three leads. Here, EGCG exhibited the highest SCORCH score compared to the other compounds (0.08712). Blind docking was also performed, as shown in Figure 2B. Although the blind docking suggested that the compounds might also bind to non-cleft regions, the SCORCH score of EGCG from blind docking was 0.03328, substantially lower than that from the focused docking. The poses obtained from focused and blind docking to the other tau fibril structures, tau-PHF-A and tau-PHF-C, are shown in Figures S4 and S5, respectively. In addition, the average SCORCH scores of the top leads binding to the three different fibril structures using focused and blind docking are summarized in Figure 3. It is clear that the EGCG has the highest SCORCH score in Tau-PHF-C of ~0.25 from focused docking compared to other fibril structures from either focused or blind docking.

To assess the significance of the score differences, we conducted independent sample *t*-tests. First, we verified its assumptions using Levene’s and Shapiro–Wilk’s tests. Samples violating these assumptions were analyzed using the Mann–Whitney U test instead. EGCG docking exhibited no significant differences between SCORCH scores in blind docking and focused docking onto tau-PHF-A (*U* = 0.0, *p* = 0.10) or tau-PHF-B (*U* = 3.0, *p* = 0.70). However, blind docking onto tau-PHF-C yielded significantly lower SCORCH scores (*M* = 0.10, *SD* = 0.01) than focused docking (*M* = 0.25, *SD* = 0.01), with a large effect size (*t*(4) = −23.20, *p* < 0.0001, *d* = −18.9462). Notably, blind docking of EGCG onto tau-PHF-C was the only blind docking scenario in which the EGCG conformer went to Site 1. A lower energy pose for EGCG on tau-PHF-C was found using focused docking compared to blind docking. CNS-11 blind docking onto tau-PHF-A (*M* = 0.01, *SD* = 0.01) had significantly lower SCORCH score than focused docking (*M* = 0.04, *SD* = 0.001), with a large effect size (*t*(4) = −8.57, *p* < 0.01, *d* = −7.00), while blind docking onto tau-PHF-C had significantly higher SCORCH scores (*M* = 0.07, *SD* = 0.01) than focused docking (*M* = 0.01, *SD* = 0.003), with a large effect size (*t*(4) = 10.62, *p* < 0.001, *d* = 8.67). The differences in SCORCH scores between blind docking and focused docking onto tau-PHF-B were not statistically significant (*U* = 2.0, *p* = 0.40). None of the CNS-11 conformers bound to site 1 when blind docking onto each tau-PHF. BHT-CNS-11 docking exhibited no significant differences between SCORCH scores in blind docking and focused docking onto tau-PHF-A (*U* = 0.0, *p* = 0.10) or tau-PHF-B (*U* = 0.0, *p* = 0.10). However, blind docking onto tau-PHF-C had significantly higher SCORCH scores (*M* = 0.09, *SD* = 0.02) than focused docking (*M* = 0.004, *SD* = 0.01), with a large effect size (*t*(4) = 7.46, *p* < 0.01, *d* = 6.09). None of the BHT-CNS-11 conformers bound to site 1 when blind docking onto each tau-PHF.

### 2.5. Binding to Amyloid Oligomers

A comparative analysis was conducted to investigate if the compounds’ SCORCH scoring would be consistent across tau fibril and oligomer targets. The protein targets used in the study include two fibrillar tau structures and three oligomer structures: one protofilament of tau-PHF-C (1taufibril), two symmetrically related protofilaments of Tau-PHF-C (2taufibril), tau monomer (1tau), tau dimer (2tau), and tau tetramer (4tau) oligomer.

**Figure 4:**
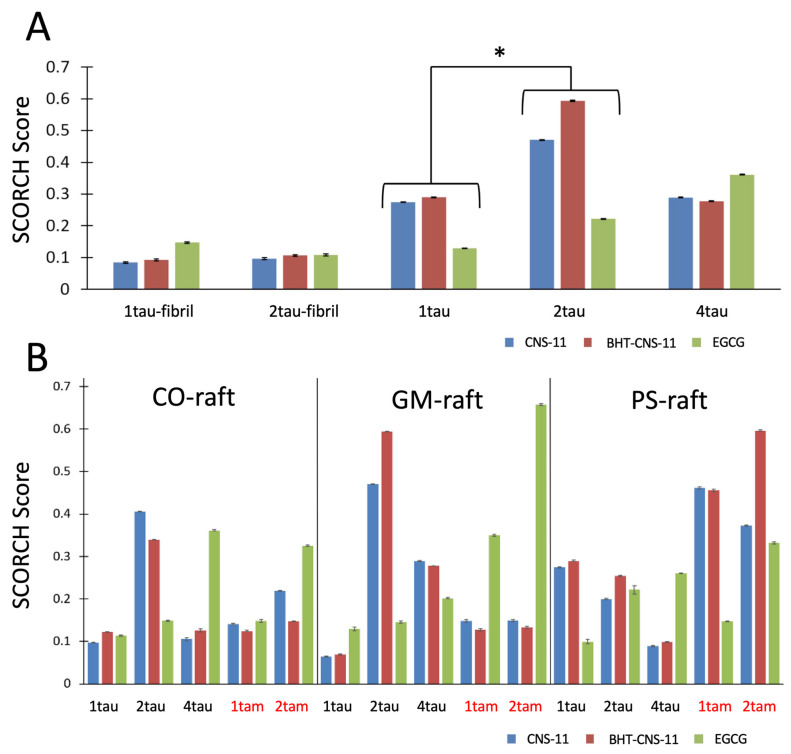
SCORCH scores of compounds docked to tau fibrils and oligomers. Average SCORCH scores of EGCG, CNS-11, and BHT-CNS-11 are displayed regardless of raft conditions comparing tau oligomers to tau fibrils and (**A**) between raft conditions between tau and tam oligomers (**B**). The mean and standard deviation of the mean (error bar) are given. Homo tau oligomers (1tau, 2tau, and 4tau) and hetero tau-amylin (1tam and 2tam) are marked in black and red, respectively. * *p* < 0.05.

In general, we observed a general trend of the compounds binding more strongly to mTCOs than to tau fibrils (Figure 4A) and the binding affinity of EGCG increased with the aggregation size of the mTCOs for either homo tau or hetero tau–amylin oligomers (Figure 4B). A detailed statistical analysis of the binding behaviors of the compounds to fibril and oligomer targets is given below.

To examine the effects of protein (i.e., 1tau, 2tau, and 4tau) and compound type (i.e., EGCG, CNS-11, BHT-CNS-11) on the SCORCH score among fibrillar and oligomeric tau structures, analysis of variance (ANOVA) was conducted, with protein as the independent variable and SCORCH pose score as the dependent variable. This analysis considers SCORCH scores for all conformers of EGCG, CNS-11, and BHT-CNS-11 across four iterations. Only the maximum SCORCH score for each compound is visualized (Figure 4A). Significant main effects were observed for the protein factor (*F*(4, 28) = 4.04, *p* < 0.05, *ηp*^2^ = 0.37), indicating general group differences in SCORCH scores across these protein structures (Figure 4A). This effect was independent of raft type and compound. However, when looking at the interaction effects between raft and protein and compound–protein in two separate two-way ANOVAs, we find that, when the raft was included, the effect of the protein on the SCORCH score was confounded (*F*(4, 28) = 2.64, *p* = 0.08, *ηp^2^* = 0.45). Still, it is important to note that the fibrils in this analysis lacked a raft condition. When the compound was included as a second factor, the effect of the protein on the SCORCH score was not confounded but retained (*F*(4, 28) = 4.00, *p* < 0.05, *ηp^2^* = 0.47). To identify the specific protein group comparisons with significantly different averages, a Tukey post-hoc test was conducted. Results show that 2tau had significantly higher SCORCH scoring compared to 1tau (*T* = −3.29, *p* < 0.05). Two other comparisons approached a significant difference, with 2tau having higher SCORCH scoring compared to 1taufibril (*p* = 0.069) and 2taufibril (*p* = 0.06). Other comparisons showed no significant difference in their means (*p* > 0.05). In summary, there was a significant effect of protein type on SCORCH scoring, and the specific subgroups of protein targets that were significant or approached significance were 1tau vs. 2tau, 1taufibril vs. 2tau, and 2taufibril vs. 2tau. Of note, 2tau consistently had the highest mean relative to the other targets across all three comparisons.

The compounds’ SCORCH scores were compared across multiple oligomeric protein targets: heterogeneous (tam) and homogeneous membrane-bound oligomers (tau and am) under different membrane-bound raft conditions (CO-, GM-, and PS-raft). A Kruskal–Wallis test was conducted, revealing significant raft effects (χ^2^(2) = 95.22, *p* < 0.0001, ε^2^ = 0.01). Post-hoc Dwass–Steel–Critchlow–Fligner (DSCF) analysis showed that SCORCH scores for PS conditions (*M* = 0.025, *SD* = 0.04) were significantly higher than for GM (*M* = 0.024, *SD* = 0.06) (*p* < 0.0001) and CO (*M* = 0.021, *SD* = 0.04) (*p* < 0.0001). DSCF post-hoc was also used to assess the differences in raft effects on individual oligomer–compound combinations. For 1tau bound to EGCG, CNS-11, and BHT-CNS-11, SCORCH scores were significantly higher for each compound in the CO-raft condition compared to the GM-raft condition (*p* < 0.001, *p* < 0.0001, *p* < 0.0001). For 1tau bound to EGCG, SCORCH scores were significantly higher in the PS-raft condition than in the CO (*p* < 0.0001) or GM (*p* < 0.0001) conditions. For the 2tau bound to CNS-11, SCORCH scores were significantly higher in the GM-raft condition than in the CO condition (*p* < 0.05) and significantly higher in the CO-raft condition than in the PS condition (*p* < 0.0001). For the 2tau bound to EGCG, SCORCH scores were significantly higher in the PS-raft condition than in the GM (*p* < 0.0001) or CO (*p* < 0.0001) conditions. For 4tau bound to EGCG, CNS-11, and BHT-CNS-11, there were no significant differences between raft conditions for each compound. For 1tam bound to BHT-CNS-11, SCORCH scores were significantly higher in the CO-raft condition than in the GM (*p* < 0.05) or PS (*p* < 0.001) conditions. For 1tam bound to EGCG, SCORCH scores were significantly higher in the PS-raft condition than in the CO (*p* < 0.001) condition. For 2tam bound to CNS-11 and BHT-CNS-11, SCORCH scores were significantly higher in the GM-raft condition than in the CO (*p* < 0.0001, *p* < 0.05) condition and higher in the PS-raft condition than in both the CO (*p* < 0.0001, *p* < 0.0001) and GM (*p* < 0.001, *p* < 0.0001) conditions. For the 2tam bound to EGCG, SCORCH scores were significantly higher in the CO-raft condition than in the PS (*p* < 0.01) condition. Overall, our results suggest that anionic lipids help promote the binding of our top leads to mTCOs.

As controls, a comparison analysis of amylin fibrils (1amylin-fibril and 2amylin-fibril), amylin monomer (1am), amylin dimer (2am), and amylin tetramer (4am) oligomers were compared to each other as well as to the above structures (Figure S6). A two-way ANOVA revealed that the subset of amylin oligomeric and fibrillar proteins had no main effects on SCORCH scoring. However, the type of compound did, with CNS-11 and BHT-CNS-11 exhibiting significantly better overall binding than EGCG (*F*(2, 30) = 6.68, *p* < 0.01, *ηp^2^* = 0.43). When running the two-way ANOVA comparing the amylin to tau-amylin oligomers, we found no significant main effects of protein or compound on the SCORCH score. However, the two-way ANOVA results for the raft and protein factor revealed a significant effect of the raft on SCORCH pose scoring (*F*(2,30) = 4.23, *p* < 0.05, *ηp*2 = 0.23), indicating general group differences in SCORCH scores across these rafts bound to the amylin and tau–amylin oligomeric protein structures. This effect was independent of compound and protein.

### 2.6. Exploring 2D and 3D Interactions between Top Leads and mTCOs

**Figure 5:**
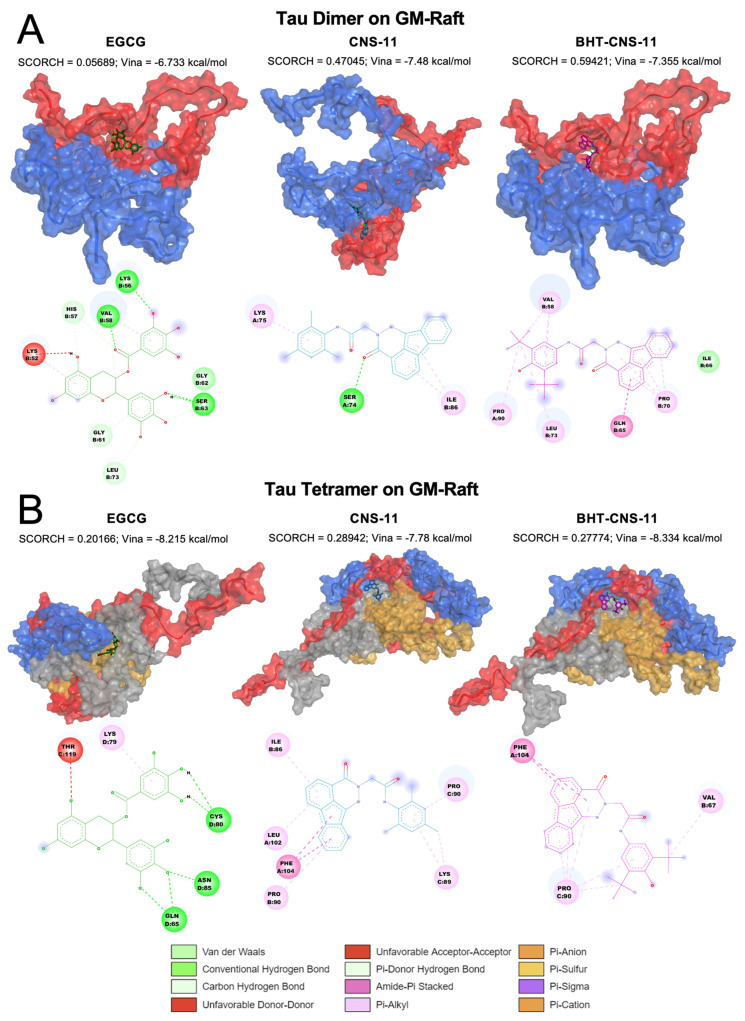
Docking of lead compounds to tau oligomers on GM-raft. The 3D structures and 2D chemical interactions of lead compounds, EGCG, CNS-11, and BHT-CNS-11, docked to tau dimer (**A**) and tetramer (**B**). The Vina and SCORCH scores are given. Tau chains are identified using different colors with chains A, B, C, and D in blue, red, gray, and orange, respectively. The chemical interactions are color coded based on the legend in the bottom.

**Figure 6:**
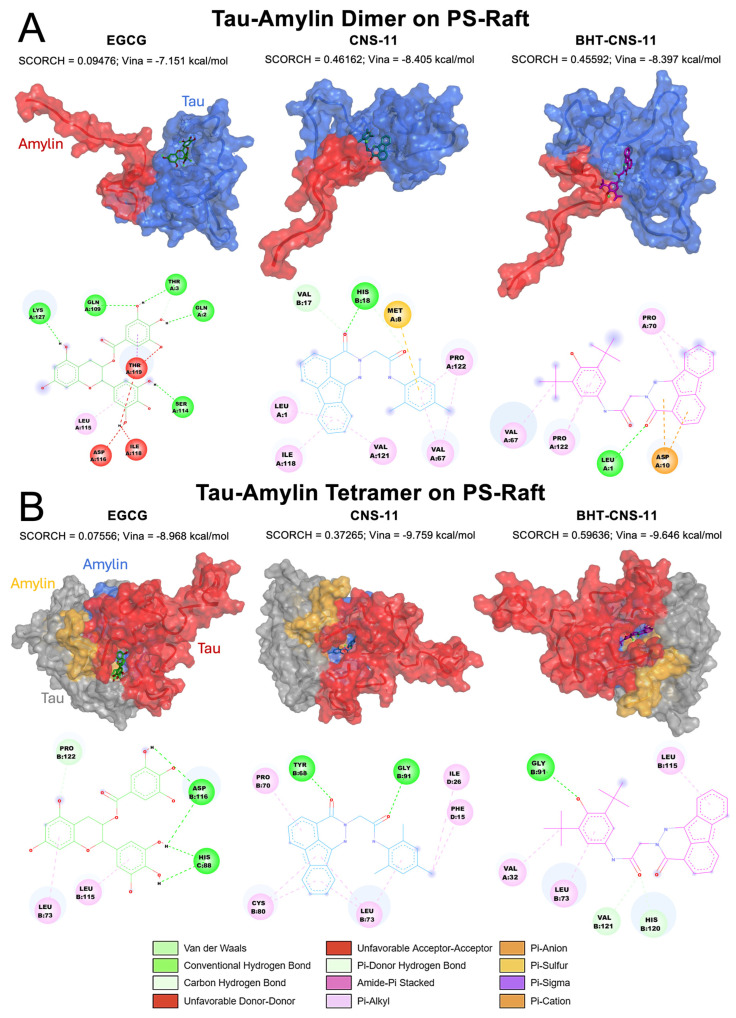
Docking of lead compounds to tau–amylin oligomers on PS-raft. The 3D structures and 2D chemical interactions of lead compounds, EGCG, CNS-11, and BHT-CNS-11, docked to tau-amylin dimer (**A**) and tau-amylin tetramer (**B**). The Vina and SCORCH scores are given. Tau and amylin chains are identified using different colors. For tau-amylin dimer, tau and amylin chains are labeled in blue and red, respectively. For tau-amylin tetramer, the tau chains are labeled in red and gray, while the amylin chains are labeled in blue and orange. The chemical interactions are color coded based on the legend in the bottom.

We observed that the top lead compounds, CNS-11, BHT-CNS-11, and EGCG, had an affinity for binding pockets within the constituent peptide chains in the middle of the amyloid oligomers. Representative 3D molecular rendering, 2D chemical interactions, and interchain binding behaviors of the compound–oligomers involving 2tau and 4tau bound to the GM-raft (Figure 5) and 1tam and 2tam bound to the PS-raft (Figure 6) are given below.

#### 2.6.1. Tau Dimer and Tetramer on GM-Raft

For the dimeric 2tau, we observed that EGCG only interacts with chain B while CNS-11 and BHT-CNS-11 interact with both chains (Figure 5A). The ketone chemical group of CNS-11 has strong hydrogen binding to Ser 74. The tri-methyl benzene and fluorene groups have strong π-alkyl interactions with Lys 75 and Ile 86, respectively. The BHT group of BHT-CNS-11 interacts with both chains A and B via alkyl and π-alkyl interactions with Val 58, Pro 90, and Leu 73. In addition, its fluorene group interacts with chain B via amide and π-alkyl interactions at residues Pro 70 and Gln 65.

For the tetrameric 4tau, we found that EGCG interacts with chains C and D, CNS-11 interacts with chains A, B, and C, and BHT-CNS-11 interacts with chains A, B, and C (Figure 5B). The fluorene group of CNS-11 has amide-π stacked interactions and π-alkyl interactions with Pro 90, Phe 104, Leu 102, and Ile 86. Its tri-methyl benzene has π-alkyl and alkyl interactions with Lys 89 and Pro 90. The fluorene group of the BHT-CNS-11 has amide-π stacked interactions with Phe 104 and alkyl–π-alkyl interactions with Pro 90. Its BHT group has alkyl and π-alkyl interactions with both Pro 90 and Val 67. The 3D oligomer–compound interactions and 2D chemical interactions between our tau dimer and tau tetramer systems from the other raft conditions, CO and PS, can be found in Figures S7 and S8, respectively.

#### 2.6.2. Tau–Amylin Dimer and Tau–Amylin Tetramer on PS-Raft

For the dimer 1tam, the structures have a propensity to bind to two different regions (Figure 6A). CNS-11 interacts with both chains A and B, while BHT-CNS-11 and EGCG only interact with chain A, which is the tau monomer of the structure. The fluorene group on the CNS-11 compound has alkyl and π-alkyl interactions with Leu1, Ile118, and Val121. Its tri-methyl group has alkyl and π-alkyl interactions with Pro 122 and Val 67, as well as a π-sulfur interaction with Met 8. The ketone group of CNS-11 has hydrogen bonding interactions with Val 17 and His 18. The BHT group of BHT-CNS-11 has alkyl and π-alkyl interactions with Val 67 and Pro 122. The ketone group of BHT-CNS-11 has hydrogen bonding with Leu1. The fluorene group on BHT-CNS-11 has a π-anion interaction with Asp10 and a π-alkyl interaction with Pro 70.

For the tetrameric 2tam, the structures have an affinity for binding to the middle area between the tau and amylin chains (Figure 6B). CNS-11 interacts with both chains B and D, BHT-CNS-11 interacts with chains A and B, and EGCG interacts with chains B and C. The ketone groups on CNS-11 have hydrogen bonding with Tyr 68 and Gly 91. Its fluorene group and tri-methyl ring have alkyl and π-alkyl interactions with Pro 70, Cys 80, Leu 73, Ile 26, and Phe 15. The ketone group of BHT-CNS-11 has hydrogen bonding interactions with Val 121 and His 120. The phenol group of BHT has hydrogen bonding interaction with Gly 91. The BHT ring, methyl group, and fluorene group have alkyl and π-alkyl interactions with Val 32, Leu 73, and Leu 115. The 3D oligomer–compound interactions and 2D chemical interactions between our tau–amylin dimer and tau tetramer systems from the other raft conditions, CO and GM, can be found in Figures S9 and S10, respectively.

#### 2.6.3. Interchain Binding Activity

Among these protein–compound interactions, we observed some interchain binding between tau chains and tau–amylin chains. Regarding their interaction with various protein secondary structures (SS), these compounds primarily interact with the random coil and occasionally with the turn. No interactions between these compounds and alpha and beta SS of the above protein structures were observed. In our analysis of the compounds bound to the 2tau, we observed that all three compounds only bind to the random coil, with some interchain interactions for CNS-11 and BHT-CNS-11 (Figure 5A). For compounds bound to 4tau, we found that they only interact with the random coil and have some interchain interactions with CNS-11, BHT-CNS-11 interacts with three chains and EGCG interacts with two chains (Figure 5B). We observed that there are some interactions with both tau and amylin monomeric chains of our 1tam and 2tam as well as binding with turn SS formation (Figure 6A). Specifically, CNS-11 binds to both tau and amylin monomeric chains of 1tam with specific interactions with amylin residues Val 17 and His 18. Additionally, this compound binds to the coil and turn at residues Val 67 and Pro 122. On the other hand, both BHT-CNS-11 and EGCG only bind to the tau monomeric chain, with BHT-CNS-11 interacting with turn SS formation at Val 67 and Pro 122 as well as with the coil. EGCG is found to only bind with the random coil. Both BHT-CNS-11 and CNS-11 bind to the interchain region between tau and amylin on the 2tam, with BHT-CNS-11 interacting with amylin at residue Val 32 and CNS-11 interacting with amylin at residues Ile 26 and Phe 15 (Figure 6B). In contrast, EGCG is found to only bind to tau chains. All three compounds were observed to bind to both random coil and turn SS, with BHT-CNS-11 interacting with turn at Leu 115, CNS-11 with Phe 15 and Ile 26, and EGCG with Leu 115 and Asp 116. The rest of the binding interactions for these compounds are with random coil secondary formation.

### 2.7. Correlation between Compound Binding and Receptor’s Secondary Structures

**Figure 7:**
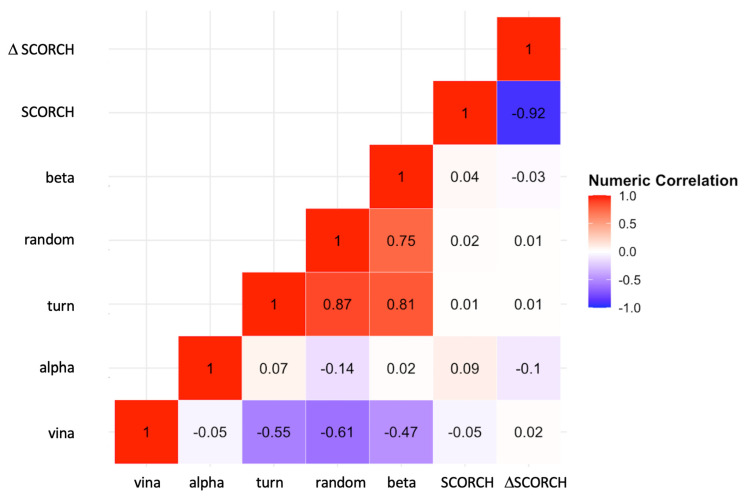
Cross-correlation matrix of docking scores of lead compounds and protein secondary structures of targets. The protein structures (alpha, turn, random, and beta) and the docking score parameters, i.e., AutoDock Vina score (vina), SCORCH score (SCORCH), and uncertainty in SCORCH score (D SCORCH), are compared. Pearson cross-correlation scores from −1.0 to 1.0 are color-coded as shown.

To further explore the correlation between binding affinity and protein secondary structures, a two-dimensional correlation Matrix (Figure 7) was constructed. This correlation matrix allows us to examine the correlations among different observables, including protein–compound Vina’s binding affinities, SCORCH scores, and secondary structures. Specifically, we aimed to investigate whether receptors containing ordered or hydrogen-bonded protein structures, such as alpha, beta, and turn, could affect the compound binding behavior compared to receptors containing disordered or non-hydrogen-bonded random structures. See Materials and Methods for a description of the calculations and classifications of secondary structures of amyloid oligomers.

Our results show no strong correlations between alpha, beta, turn or random, and SCORCH scoring. However, Analysis of Covariance (ANCOVA) indicated a significant effect of the ordered turn structure on the SCORCH score across all amyloid oligomers. The effect of protein structure on SCORCH score in ANCOVA remained significant compared to one-way ANOVA (*F*(7, 64) = 2.50, *p* < 0.05, *ηp*^2^ = 0.21) when controlling for the turn covariate (*F*(7, 63) = 3.36, *p* < 0.01, *ηp*^2^ = 0.27). Additionally, the turn covariate demonstrated significant effects when controlling for the protein variable (*F*(1, 63) = 5.80, *p* < 0.05, *ηp*^2^ = 0.08), suggesting that protein effects may vary depending on the level of turn. ANCOVA also showed that protein effects on SCORCH scores were not retained after introducing beta secondary structure as the covariate (*F*(7, 63) = 1.93, *p* = 0.08, *ηp*^2^ = 0.18), indicating that beta may account for some of the variances in the SCORCH scores that were previously attributed to the protein factor in one-way ANOVA. There was a strong negative correlation between both the turn structure (*r* = −0.6) and the random structure (*r* = −0.6) with Vina’s binding affinity, but not for the other alpha and beta structures. Interestingly, we discovered a weak correlation (*r* = −0.05) between Vina’s Binding Affinity and SCORCH score.

## 3. Discussion

In this study, we used an enhanced ML approach called SCORCH to predict the potential binding of selected compounds to experimentally derived tau fibrils and in silico mTCOs from physics-based MD simulations [18,32]. The SCORCH poses rescoring algorithm effectively re-ranked compound conformers based on an ML model of 487 features selected for optimal SF performance [42]. As a result of SCORCH implementation, we obtained increased accuracy and time efficiency in identifying competitive multipurpose compounds, providing insight into specific protein–compound interactions necessary for therapeutic intervention. Overall, through the use of ML-enhanced rescoring of selected compounds and MD simulations of oligomeric structures, we have confirmed the specific binding of EGCG onto the EGCG-binding site (Site 1) from Cryo-EM and proposed alternative binding sites for CNS-11 and BHT-CNS-11 in both fibrils and mTCOs. Additionally, we have identified binding interactions between tau fibril-disaggregating compounds and mTCOs under different lipid raft conditions. In order to align our approach with existing knowledge on tau amyloidogenesis, we analyzed compound binding interactions to tau monomers, dimers, and tetramers after binding to intracellular PS-containing raft and extracellular GM-containing raft. The variety of tau structures we have used in this study allows us to capture the dynamic binding interactions between our compounds and tau in different polymorphisms and environments.

To ensure a comprehensive analysis, we employed both blind and focused docking approaches. The top CNS-11 and BHT-CNS-11 compound poses exhibited binding outside of Site 1 of both tau-PHF-B and tau-PHF-A when using blind docking. Blind docking of our compounds onto tau-PHF-C revealed that the top EGCG compound conformers bound to Site 1 with higher scores and stronger binding compared to the other fibrils, but the binding pocket was consistent across fibrillar structures. However, blind docking to tau-PHF-C showed that the top CNS-11 and BHT-CNS-11 compound conformers had a better binding affinity and higher SCORCH score outside Site 1. We propose the investigation of alternative binding sites other than Site 1 as these may be easily targeted by CNS-11 or BHT-CNS-11. This computational evidence suggests that compound SCORCH scores are contingent on the docking approach (i.e., focused or blind). An additional contributing variable may be whether the receptor structure is in Apo- or Holo-state. However, because Site 1 is an experimentally confirmed binding site [13], the compound poses obtained through blind docking may warrant further experimental and computational investigation. Importantly, EGCG is shown to have amyloid fibril-disaggregating properties but is unselective in its approach [13], meaning that our other compounds, namely CNS-11 and BHT-CNS-11, should be further experimentally investigated for selectivity of specific amyloid types. Overall, our computational findings highlight the comprehensive benefits of utilizing blind and focused docking.

Regarding the protein–compound chemical interactions at the protein residue level, we found EGCG on tau-PHF-C to be consistent with EGCG on tau-PHF-B from the aforementioned study, namely, residues Asn 327, His 329, and Glu 338 [13]. This indicates that these interactions may be critical for the stability of the compound in this protein’s binding pocket and for destabilizing the aggregated protein. Stemming from this, we hypothesized that EGCG would bind to the same residues in our tau and tau–amylin oligomers through similar chemical interactions. Contrary to our hypothesis, we found that, among our top protein–compound SCORCH scorers, our top three compounds bind to the R2 and R3 regions of 2tau, and R3 and R4 regions of 4tau: these were different residues than for EGCG binding on tau-PHF [13]. Differential binding of EGCG between oligomers and fibrils suggests a possible distinct mechanism of disaggregation. When CNS-11 binds to 2tau, its ketone group binds to Ser 74 of chain A. This is one of the residues that is targeted by kinases during phosphorylation, causing tau’s dissociation from microtubules [14]. This differs from the charge-pairing mechanism suggested to disaggregate tau fibrils by repelling charges between Glu amino acids [13]. By binding to this phosphorylation site of the tau oligomer, CNS-11 may exhibit capabilities of modulating tau phosphorylation in other phases of tau amyloidogenesis. Despite the need for further experimental validation, our findings reveal the residue-specific interactions between compounds and proteins and propose inhibition of phosphorylation as a potential mechanism of CNS-11. Since seeding-competent tau oligomers are known to occur before neurofibrillary tangles (NFTs) form in the brain [19], and because AD progresses slowly but worsens rapidly [56], identifying the residue contacts and potential mechanism of action of compound–oligomer interactions are crucial for the treatment of AD.

Using ML and MD simulations, we predict that EGCG, CNS-11, and BHT-CNS-11 can bind more strongly to mTCOs than to tau fibrils. This could be due to various reasons, including the addition of R1 and R2 tau repeats to the tau molecules in the mTCOs, the increased number of binding pocket cavities on the surface or inside the protein for compound binding, increased hydrogen bonding ability, or interaction with lipid raft. This suggests that these compounds may be more likely to bind to the 4R isoform of tau. This isoform is prevalent in the early stages of AD. It occurs before assembly into the higher molecular weight and more organized PHF than the tau-PHF that is observed in the later stages of AD. Previous research has shown that R2 and R3 tau repeats form seed-competent fibrils in the presence of heparin, suggesting that these compounds may be important in blocking the self-aggregation and seeding potency of tau [26].

Tau is a very polymorphic protein whose role in neurotoxicity in AD can not be confined to just one structural conformation. ML-based MD trajectory clustering can be used to cluster the frames or conformations of the proteins from the trajectory file into groups based on their structural similarities [57]. In this paper, we utilized Ward’s method for hierarchical cluster analysis to compute all pairwise RMSD values between conformations and then determined clusters with minimum variance. These clusters are shown in Figure S11. A representative protein is derived from each cluster for use in molecular docking to determine the consistency and stability of the compound binding to multiple conformations of the protein. The binding of CNS-11 to 2tau on GM-raft consistently exhibited interchain binding to the same protein area facing the lipid interface across different protein conformations, as shown in Figure S12. Additionally, the CNS-11 fluorine group consistently binds to Pro 70 and Leu 73, which correspond with Pro 332 and Leu 315 in the full-length 441 residue tau. In previous research, these residues have been defined as membrane-anchoring sites to the GM-containing raft [18]. This opens the possibility of CNS-11 disrupting any protein–lipid interactions that occur in this system. Note that tau dimer has been found to induce membrane disruption in GM-raft [32]. Therefore, administering CNS-11 may block this specific protein–lipid interaction. Future work will include further analysis of binding consistency across multiple conformations of in silico structures.

Additionally, our top three compounds bind to R1, R3, and R4 regions of tau on the dimeric 1tam, and only CNS-11 interacts with the amylin chain [18]. The top three compounds bind to R3 and R4 regions of tau on the tetrameric 2tam, but of our three compounds, only BHT-CNS-11 and CNS-11 interact with amylin chains. Importantly, none of the compounds interacted with the residues on the tau and tau–amylin structures that EGCG interacted with in the tau-PHF systems. More specifically, the CNS-11 binds to Val 121 of 1tam, and BHT-CNS-11 binds to Val 121 of 2tam, previously defined as key hydrophobic residues of the tau chains for membrane binding to PS-raft [32]. Additionally, CNS-11 interacts with both tau and amylin chains of the heterogeneous structure, potentially blocking tau-inducing alpha-to-beta refolding [32]. Of the key hydrophobic residues in the amylin component of our heterogeneous structures, BHT-CNS-11 was found to bind to Val 32, and CNS-11 bound to Ile 26: two of the major membrane anchoring sites of both 1tam and 2tam to PS-raft [32]. Interestingly, CNS-11 was also found to bind to Cys 80 on 2tam, which corresponds to Cys 322 on the full-length 441 residue tau. This residue is important for interactions between tau molecules or other proteins via thiol-disulfide exchange and contributes to the formation of dimers and granular oligomers [58]. Additionally, substitution of this residue with alanine affects the self-aggregation and seeding activity of tau [59]. EGCG did not exhibit any binding to key hydrophobic residues of either tau or amylin components of our hetero tau–amylin oligomers. This indicates that the CNS-11 compound may destabilize interactions between the tau–amylin oligomers and lipids on the PS-raft by interacting with amylin zones that anchor the protein to the membrane [32].

Our compounds demonstrated preferential binding towards dimeric homogeneous tau structures compared to tau monomers and fibrils. Using Tukey post-hoc comparison, we evaluated the statistical significance of the differences between average SCORCH scores across all three ligands and their conformers between each protein target. This analysis aggregated all of the surfaces and protein target replicates. When comparing the overall SCORCH score of the top three compounds to tau fibrils and oligomers, we discovered that 2tau has a significantly higher score relative to 4tau and approaches a statistically significant difference compared to both 1taufibril and 2taufibril. Past research found a similar trend, where the interaction energy between tau and DLPC was stronger in 2tau compared to 4tau and 1tau in the GM-raft condition, which could have caused a conformational change in the protein that made compound binding more favorable [32].

Additionally, a comparison across the top three compounds bound to tau and tau–amylin oligomers revealed significantly higher average SCORCH scores in the PS-raft condition compared to the CO- or GM-raft conditions. The DSCF post-hoc analysis was used to assess the statistical significance of the differences between average SCORCH scores across all three ligands and their conformers between each raft condition. This analysis aggregated all of the oligomeric protein targets and their replicates. Tau fibril could not be included in this analysis as it did not have a raft condition. Past research found that the protein–protein interchain energy in heterogeneous 2tam was stronger in the PS-raft than in the GM-raft. Additionally, the interaction energy between the mTCOs and GM1 of the GM-raft was stronger than with the POPS in the PS-raft. The increased interaction energy with GM1 of the GM-raft may have caused a conformational change that created unfavorable compound binding with the protein compared to the PS-raft [32]. A previous study reported increased SS formation of homogeneous and heterogeneous oligomers bound to the PS- and GM-raft as detected in MD simulation compared to CO-raft [32]. Notably, mTCOs bound to PS-raft have a higher number of amino acids with beta secondary structure than those bound to GM-raft, indicating a possible effect of beta protein–compound binding, as we discussed in our ANCOVA analysis. Additionally, a three-way interaction between protein–protein and protein–lipid is suggested, as past research shows that the cross-seeding event between tau and amylin causes an increase in alpha helix formation in the PS-raft condition and an increase in beta-sheet formation in the GM-raft condition. These findings are consistent with previous lines of research suggesting that PS- and GM-functional nanodomains, when interacting with early amyloidogenic aggregates, regulate membrane damage and protein aggregation [25]. Interestingly, our statistically significant findings were PS-raft dependent, counter to previous studies, including GM-raft. One of the reasons may be that because the PS-raft has the propensity to induce alpha-helices formation in the hetero-oligomers and higher beta-sheet formation in all mTCOs relative to GM-raft, compound conformers might have a stronger affinity for protein structures rich in both alpha helices and beta sheets. Unlike the most current treatment strategies in clinical trials targeting synaptic tau oligomers, our targets allow us to explore intracellular tau oligomers prior to their release [60]. Through this analysis, we identified oligomers from the PS-raft condition as feasible targets.

Targeting tau in its oligomeric form may reduce the possibility of disaggregated products becoming toxic. Future directions for our work include utilizing tools to improve the blind docking approach for detecting the best compound conformation and identifying the capacity of specific compounds to destabilize specific secondary structures such as alpha helices and beta sheets characteristic of tau and tau–amylin oligomer aggregation [32]. For example, a fragment docking protocol has been developed to improve the blind docking performance of Vina [61]. This is a predictive tool with a full-scale approach to the protein’s surface, systematically searching for smaller simulation boxes to which the compound might bind. Rather than using a sole blind docking approach in which the native compound conformation might be lost, this approach would enable effective identification of the compound binding site.

The methodology outlined here comes with its limitations. In terms of which target to use, this study primarily tested binding to the last frame of the MD-simulated oligomers. It is important to note that proteins take multiple conformations when bound to a membrane. So, establishing the time-dependent docking technique to observe the consistency of compound docking to multiple conformations (or frames) of the oligomers is important in determining the feasibility of the compound affecting the oligomers when bound to a membrane containing GM and PS. Additional practices such as MD simulations of the oligomers bound to the compound, e.g., tau-Random Acceleration MD advanced sampling [62], and molecular docking onto oligomers after interacting with oxidized membranes are necessary to observe not only how well the compound interacts with and stays bound to the oligomers, but also how such compounds can affect tau oligomers that interact under various membrane environments. With these considerations, we attempt to retain the flexibility that is essential to experimentation with such unpredictable structures.

In drug discovery, it is important to test large datasets of compounds and to practice other molecular docking procedures, such as pocket searching. In this paper, we primarily focus on select compounds derived from previous experimentation and blind-docking methods. Other considerations are compound-based searching methods and employing grid box pocket searching software. Through chemoinformatic tools such as RDKit [63], we are able to perform a compound similarity search to search for structures with target properties, drug-likeness, and pre-defined Tanimoto similarity scores. With these considerations, we continue to explore promising avenues for identifying novel therapeutic candidates not only targeting AD and type 2 diabetes but also other diseases characterized by aggregated protein structures.

## 4. Materials and Methods

### 4.1. Compounds

Compounds were chosen with the following properties in mind: antioxidant, anti-aggregation, and enzyme inhibition. BHT-6QH is observed to inhibit GSK-3β and has antioxidant properties [43]. BHT-E20, a hybrid of BHT and Donepezil or E20, is observed to inhibit AChE and has antioxidant properties [49]. EGCG has been observed to disaggregate Tau-PHF [13] and exhibits antioxidant properties [37] but has low bioavailability [13]. CNS-11, structurally similar to RGCG, has also been found to disaggregate tau-PHF, but no antioxidant or enzyme inhibitory properties have been observed [13]. BHT-CNS-11 is a new compound containing both the disaggregating components of CNS-11 and the antioxidant BHT group (Table 1).

### 4.2. Cheminformatics Tools for Screening Compound Druggability

The chemical absorption, distribution, metabolism, excretion, and toxicity or ADMET properties and bioactivity scores were predicted using the online and open-source predictive model of ADMET properties, pkCSM [51] and ADMETLab 3.0 [52]. ADMETLab 3.0 was used to evaluate the Log P and Log D values, as these measurements were not available in the pkCSM program. Molecular properties and drug-likeness scoring were evaluated using Molsoft (https://www.molsoft.com/servers.html, accessed on 5 June 2024). The predictions are described below.

For absorption, the proportion of compounds to be absorbed through the human small intestine after oral administration was predicted. In addition, a compound must also be soluble to ensure good absorption. Predicted water solubility reflects the solubility of the compound in water at 25 °C using experimental water solubility measurements of 1708 compounds. Log P was predicted based on the log of the concentration of the compound in the octanol phase divided by the concentration of the compound in the water phase of 12,682 compounds [52]. Log D was predicted based on the log of the concentration of the compound in the octanol phase divided by the sum of the concentration of the ionized compound and non-ionized compound in the water phase of 19,155 compounds [52]. The fractional lipophilicity index (FLI) was calculated by taking the product of 2 and Log P and subtracting it from the absolute value of Log D at pH = 7.4 [54].

To enable distribution analysis, pkCSM constructed a predictive model for steady-state volume of distribution (VDss) using data from 670 compounds tested in humans. Similarly, the Blood–brain permeability predictive model was developed using the logarithmic ratio of plasma compound concentrations (log BB) from 320 compounds in animal models. Furthermore, the predictive model for CNS permeability was derived from in situ brain perfusions that tested 153 compounds directly injected into the carotid artery.

For metabolism, the predicted levels of inhibition to Cytochrome P450 enzyme isoforms induced by each molecule were based on data from over 14,000 compounds with cytochrome P450-inhibition qualities. Compounds that inhibit these enzymes can affect compound metabolism. Additionally, the metabolism of the compounds was predicted based on previous data calculating the metabolism induced by each isoform of cytochrome P450 on 671 compounds.

For excretion, the amount of compound cleared from the hepatic and renal system over time was predicted based on previous data calculating the hepatic and renal clearance of 398 compounds. This can be used to determine the dosing rates and steady-state concentrations.

For toxicity, the predicted maximum recommended toxic dose threshold (MRTD) was based on previous data measuring the MRTD of 1222 compounds in human clinical trials. This measurement is used to guide phase I clinical trials to determine which dose to start with. Additional measurements of toxicity included the AMES test, hERG I and II inhibition, and hepatotoxicity. AMES toxicity predicts a compound’s mutagenic potential based on previous bacterial assays of over 8000 compounds. The hERG I and II inhibition predictive model was developed based on the hERG inhibition qualities of 368 and 806 compounds, respectively. Strong inhibitors of hERG can cause cardiotoxic side effects. This model primarily looks at whether a compound is likely to inhibit hERG, but it does not measure the level of inhibition. Compound-induced liver injury, or hepatotoxicity, was predicted based on previously measured side effects of 531 compounds.

### 4.3. Experimental Protein Structures

Atomistic structures of three Tau fibrils were obtained from previously published Cryo-EM structures from the brain autopsies of AD patients [11,13]. Here, tau-paired helical fibrils (PHF) were calculated from the Cryo-EM images. In this study, tau-PHF-A (PDB: 7UPE/EMD-26663) and tau-PHF-B (PDB: 7UPE/EMD-26663) refer to the Cryo-EM structures in the absence and presence of EGCG [13], and Tau-PHF-C (PDB: 5O3L) refers to another Cryo-EM structure in the absence of EGCG from another source [11]. All three Cryo-EM structures exhibit two pentameric fibrils interacting at the loop regions of the pentamers. Tau-PHF-B has a well-defined cleft region where a stack of EGCG molecules was located in the Cryo-EM image. The EGCG in tau-PHF-B was removed for compound docking and is considered a HOLO-structure for docking. Neither tau-PHF-A nor tau-PHF-C contain bound compounds and are considered APO structures for docking. Note that in addition to tau PHF-fibrils, Cryo-EM amylin PHF-fibrils (PDB: 6VW2) given by Cao et al. [50] were also used for docking and the results compared with tau fibril binding. The much smaller amylin fibrils were derived from recombinant amylin proteins.

### 4.4. In Silico Membrane-Bound Amyloid Oligomers

In silico membrane-bound amyloid oligomers were created using physics-based coarse-grained (CG) and atomistic (AA) MD simulations [18]. We successfully designed and simulated various self-assembled amyloid oligomers of sizes up to tetramers in solution. These soluble oligomers bind to mimics of the cytoplasmic and exoplasmic leaflets of the neuronal plasma membrane containing anionic PS and GM1 lipids, or PS-raft and GM-rafts, respectively, within a few microseconds. The simulated membrane-bound tau oligomers were stable for up to 5 microseconds in the CG time scale and exhibited surface-induced protein folding on both PS- and GM1-containing membranes in the AA time scale. As controls, the binding of amyloid oligomers to raft membranes without anionic lipids, or CO-raft, was also performed. The membrane-bound amyloid oligomers remained stable after 100 ns of AA simulations. The last 100 ns of the AA structure of each amyloid oligomer was extracted and served as the in silico amyloid oligomer receptor for this docking study. Details of multiscale MD simulations of amyloid oligomers can be found elsewhere [18,32,40]. In this study, behaviors of compound binding to our in silico membrane-bound tau-containing oligomers, or mTCOs, were compared with those of the Cryo-EM-derived tau fibrils. Both homo tau and hetero tau–amylin oligomers were examined. Since amylin is involved in the hetero-oligomers, additional investigation was also performed for compound binding to the in silico homo amylin oligomers and Cryo-EM amylin fibrils. Note that the Cryo-EM structure of amylin fibrils contains no bound compound, as opposed to the EGCG-bound tau-PHF-B.

The protein secondary structures of all amyloid oligomers in this study were calculated from the AA simulation using the Define Secondary Structure of Proteins algorithm [64]. Here, we grouped the seven resolved structures into three subgroups, beta, alpha, and random, while turn remained an independent group. Specifically, the beta group includes beta-sheet and beta-bridge, the alpha group includes A-helix, 5-helix, and 3-helix, and random includes bend and coil. Note that beta, alpha, and turn are ordered, involving hydrogen bonding between residues, while random is non-ordered, with no hydrogen bonding between residues.

### 4.5. Molecular Docking

AutoDock Vina (v 1.2.4) was used for computational docking of the protein and compound [41] and the Scoring Consensus for RMSD-based Classification of Hits (SCORCH) ML program [42] was used for re-ranking compound conformers. For the preparation of the protein structures, Autodock Tools (v 1.5.7) [41] was used to remove any water, ions, lipids, or heteroatoms, to add hydrogen atoms, and to assign Gasteiger charges [65]. Marvin Sketch from ChemAxon (Boston, MA, USA) was used to calculate the protonation state of each compound at a pH of 7.4 and to generate a 3D structure. OpenBabel [66] was used to enumerate tautomeric forms of our compounds and generate 3D conformers. A grid spacing of 1.00 Angstrom, maximum energy difference from the best pose of 3 kcal/mol, number of modes of 20, and exhaustiveness of 32 were used for the AutoDock Vina parameters. For blind docking, the search box parameters were set to encapsulate the full protein structure. For focused docking of Tau-PHF structures, we used a search box 20 Å × 16 Å × 12 Å in size. All oligomeric structures were subject to blind docking, assuming no known pockets. Docking methods typically assume that there is prior knowledge of the protein holo-structure (bound). This assumption does not have much real-world application, so we evaluated methods with imaged HOLO and APO (unbound) fibril structures, as well as computationally generated APO structures of our in silico amyloid oligomers. Visualization of the protein–compound 2D chemical interactions was performed using BIOVIA Discovery Studio Visualizer v21 (San Diego, CA, USA). Both Discovery Studio and VMD v1.9.4 [67] were used to generate 3D visualizations of the protein–compound and lipid–protein–compound visualizations.

### 4.6. ML-Enhanced Re-Scoring

SCORCH was used to rescore the 20 poses output by Autodock Vina to identify the best pose for each compound and protein. SCORCH was used only to re-score the already docked compounds against the protein target. It was not used for molecular docking itself. All 8 compounds and their poses were evaluated for each protein target with the SCORCH parameters for threads set to 6. Molecular docking with AutoDock Vina and subsequent SCORCH re-scoring was repeated for a total of 4 iterations to increase the sample size for data analysis. The SCORCH program utilizes both atomic interactions and binding analysis tool, BINANA [68], features observed between experimental proteins and compounds from database packages, PDBbind, BindingMOAD, and Iridium, to assign the predicted strength in protein–compound binding of new protein–compound pairs (SCORCH score) [42]. In this work, higher SCORCH scores are assigned to stronger binders. This ML-based algorithm provides improved pose identification of compound–target complexes by successfully determining which features drive strong protein–compound binding.

## 5. Conclusions

Using ML-based SCORCH and MD simulations, we have demonstrated that EGCG, CNS-11, and BHT-CNS-11 bind more strongly to mTCOs than to tau fibrils. The SCORCH scoring function of EGCG binding to mTCOs increases with the oligomer aggregation size, while those of CNS-11 and BHT-CNS-11 show a preference for the dimeric mTCOs over the tetrameric mTCOs, suggesting that an intracellular oligomeric target is feasible for future discovery of new therapeutic interventions. The presence of anionic lipids, particularly PS, promotes the binding of the compounds to mTCOs. Future studies should utilize both computational and experimental methods to explore compound binding consistency across different protein conformations, compound resident time, and the protein-folding changes induced by each compound. Given the scarcity of protocols and experimental tools for creating and imaging the membrane-bound soluble intermediate forms of tau or heterogeneous tau–amylin in vitro and in vivo, molecular docking to these in silico MD-derived structures is the closest approach for examining residue-level interactions with different compounds and advancing future AD drug development. The strength of the binding interactions of these compounds can be confirmed via molecular dynamics simulations, and the protein conformational changes they induce can be observed in a physiologically relevant environment.

## Figures and Tables

**Table 1:** Structure–activity relationships of small molecules targeting tauopathies.

Compound	Structure	Activities
6QH	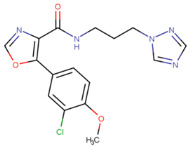	GSK-3β inhibition ^1^
E20	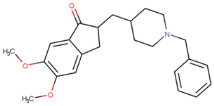	AChE inhibition ^2^
BHT	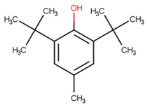	Antioxidation ^3^
EGCG	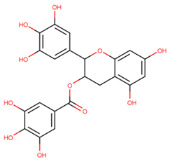	Antioxidation + Tau fibril disaggregation ^4^
CNS-11	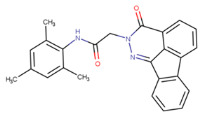	Tau fibril disaggregation ^5^
BHT-6QH	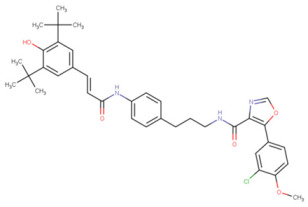	Antioxidation + GSK-3β Inhibition ^6^
BHT-E20	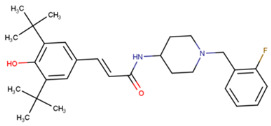	Antioxidation + AChE inhibition ^7^
BHT-CNS-11	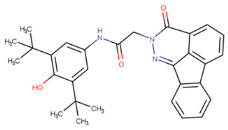	Antioxidation + tau fibril disaggregation ^8^

^1^ An isoform of glycogen synthase kinase 3 (GSK3) linked to tau phosphorylation in tauopathy [43]. ^2^ Acetylcholinesterase (AChE) linked to degraded acetylcholine (AChE) [44]. ^3^ Eliminates free radicals caused by oxidative stress [38]. ^4^ Antioxidation and tau fibril disaggregation as shown in Seidler et al. [13]. ^5^ Tau fibril disaggregation as shown in Seidler et al. [13]. ^6^ Antioxidation and GSK-3b inhibition as shown in Luo et al. [43]. ^7^ Antioxidation and AChE inhibition as shown in Cai et al. [49]. ^8^ Predicted antioxidation and tau fibril disaggregation as proposed in this study.

**Table 2:** Tau-containing receptors for anti-aggregation and antioxidation drug development.

Type	Name	Structure	Source
Fibril (Cryo-EM)	Tau-PHF-A	2 Pentamers	[PDB ID 7UPE/EMD-26663] ^1^
Fibril (Cryo-EM)	Tau-PHF-B	2 Pentamers with EGCG	[PDB ID 7UPF/EMD-26665] ^2^
Fibril (Cryo-EM)	Tau-PHF-C	2 Pentamers	[PDB ID 5O3L] ^3^
Fibril (Cryo-EM)	Amylin-PHF	2 Pentamers	[PDB ID 6VW2] ^4^
Tau Oligomers (In Silico)	1tau 2tau 4tau	Monomer Dimer Tetramer	Homo tau oligomers on CO-, PS, and GM-raft membranes ^5^
Amylin Oligomers (In Silico)	1am 2am 4am	Monomer Dimer Tetramer	Homo amylin oligomers on CO-, PS-, and GM-raft membranes ^6^
Tau–Amylin Oligomers (In Silico)	1tam 2tam	Dimer Tetramer	Hetero tau–amylin oligomers on CO-, PS-, and GM-raft membranes ^7^

^1^ Cryo-EM structure from human AD brain autopsy tissues as given by Seidler et al. [13]. ^2^ Same as above but with samples incubated with EGCG for 3 h as given by Seidler et al. [13]. ^3^ Cryo-EM structure from human AD brain autopsy tissues as given by Fitzpatrick et al. [11]. ^4^ Cryo-EM structure from human AD brain autopsy tissues as given by Cao et al. [50]. ^5^ In Silico structure from the last 100 ns of multiscale MD simulations as given by Cheng et al. [18]. ^6^ In Silico structure from the last 100 ns of multiscale MD simulations as given by Nguyen et al. [40]. ^7^ In Silico structure from the last 100 ns of multiscale MD simulations as given by Santos et al. [32].

## Data Availability

The data presented in this study are available in article and Supplementary Materials.

## Supplementary Materials

The following supporting information can be downloaded at: https://www.mdpi.com/article/10.3390/molecules29122818/s1, Figure S1: Absorption properties of compounds; Figure S2: Distribution properties of compounds; Figure S3: Excretion and toxicity properties of compounds; Table S1: Metabolic inhibition of compounds; Table S2: Toxicity of compounds; Table S3: Molsoft-prediction of molecular properties and drug-likeness of compounds; Figure S4: Docking of compounds to tau-PHF-A; Figure S5: Docking of compounds to tau-PHF-C; Figure S6: Docking of compounds to amylin fibrils and oligomers; Figure S7: Docking of lead compounds to tau oligomers on CO-raft; Figure S8: Docking of lead compounds to tau oligomers on PS-raft; Figure S9: Docking of lead compounds to tau–amylin oligomers on CO-raft; Figure S10: Docking of lead compounds to tau–amylin oligomers on GM-raft; Figure S11: Hierarchical clustering dendrogram of time-dependent tau dimer structures; Figure S12: Docking of compounds to tau dimer structures from different time frames.

## Abbreviations

AD	Alzheimer’s disease
mTCOs	Membrane-bound tau-containing oligomers
EGCG	Epigallocatechin-3-gallate
BHT	Butylated hydroxytoluene
MD	Molecular dynamics simulations
ML	Machine learning
PS	Phosphatidylserine
GM1	Monosialotetrahexosylganglioside
PS-raft	Lipid raft containing PS mimicking cytoplasmic neuronal membrane leaflet
GM-raft	Lipid raft containing GM1 mimicking exoplasmic neuronal membrane leaflet
CO-raft	Lipid raft without anionic lipids mimicking non-specific neuronal membrane leaflet
SCORCH	Scoring consensus for RMSD-based classification of hits
GSK-3β	3β isoform of glycogen synthase kinase
AChE	Acetylcholinesterase
PHF	Paired-helical fibril
ADMET	Absorption, distribution, metabolism, excretion, and toxicity
FLI	Fraction lipophilicity index
BBB	Blood–brain barrier
VDss	Steady-state volume of distribution
MRTD	Maximum recommended toxic dose threshold
1am	Monomeric amylin
2am	Dimeric amylin in homo-amylin oligomer
4am	Tetrameric amylin in homo-amylin oligomer
1tau	Monomeric tau
2tau	Dimeric tau in homo-tau oligomer
4tau	Tetrameric tau in homo-tau oligomer
1tam	Dimeric tau–amylin oligomer
2tam	Tetrameric tau–amylin oligomer
ANOVA	Analysis of variance
DSCF	Dwass–Steel–Critchlow–Fligner
ANCOVA	Analysis of covariance
RMSD	Root mean square deviation
AA	All-atom
CG	Coarse-grained
τ-RAMD	Drug-target residence time (τ)-random acceleration MD
RDKit	Open-source cheminformatics and machine-learning software for drug discovery
