# Supplementary material for: Exploring Tau Fibril-Disaggregating and Antioxidating Molecules Binding to Membrane-Bound Amyloid Oligomers Using Machine Learning-Enhanced Docking and Molecular Dynamics
Source: Molecules. 2024 Jun 13;29(12):2818. doi: 10.3390/molecules29122818 (PMC11206291; doi:10.3390/molecules29122818)
Supplement: Supplementary file 1 [file molecules-29-02818-s001.zip › molecules-3041209-supplementary.pdf]

Supporting Information

Exploring Tau Fibril-Disaggregating and Antioxidating Molecules Binding to Membrane-Bound Amyloid Oligomers Using Machine Learning-Enhanced Docking and Molecular Dynamics

Table of Contents

Figure S1. Absorption, water solubility, and lipophilicity properties of compounds.
Figure S2. Distribution properties of compounds
Figure S3. Excretion and Toxicity of compounds.
Table S1. Metabolic inhibition of compounds.
Table S2. Toxicity of compounds.
Table S3. Molsoft-prediction of molecular properties and drug-likeness of compounds.
Figure S4. Docking of compounds to tau-PHF-A.
Figure S5. Docking of compounds to tau-PHF-C.
Figure S6. Docking of compounds to amylin fibrils and oligomers.
Figure S7. Docking of lead compounds to tau oligomers on CO-raft.
Figure S8. Docking of lead compounds to tau oligomers on PS-raft.
Figure S9. Docking of lead compounds to tau-amylin oligomers on CO-raft.
Figure S10. Docking of lead compounds to tau-amylin oligomers on GM-raft.
Figure S11. Hierarchical clustering dendrogram of time-dependent tau dimer structures.
Figure S12. Docking of compounds to tau dimer at selected time frames.

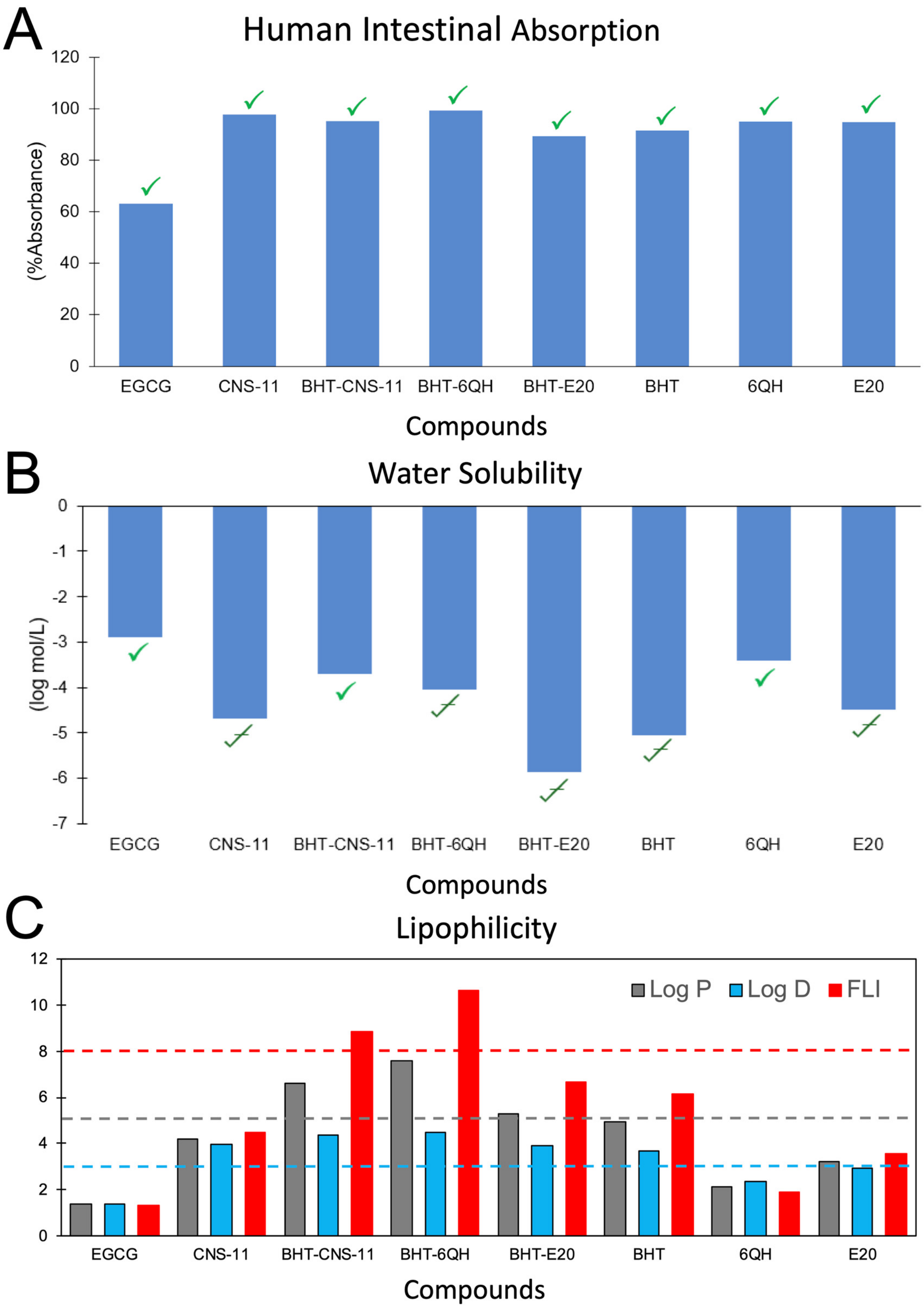

Figure S1. Absorption, water solubility, and lipophilicity properties of compounds EGCG, CNS-11, BHT-CNS-11, BHT-6QH, BHT-E20, BHT, 6QH, and E20. The human intestinal absorption in terms of % absorbance (**A**) and water solubility in terms of log mol/L (**B**) were predicted by pkCSM with check marks indicating optimal values for bioavailability and half check marks indicating moderate values. The thresholds and definitions of human intestinal absorption and water solubility can be found in the main text. The lipophilicity (**C**) was predicted by ADMETlab. The definitions of Log P, Log D, and fractional lipophilic index (FLI) can be found in the main text, and the upper thresholds of these lipophilic parameters are given in dashed lines, respectively.

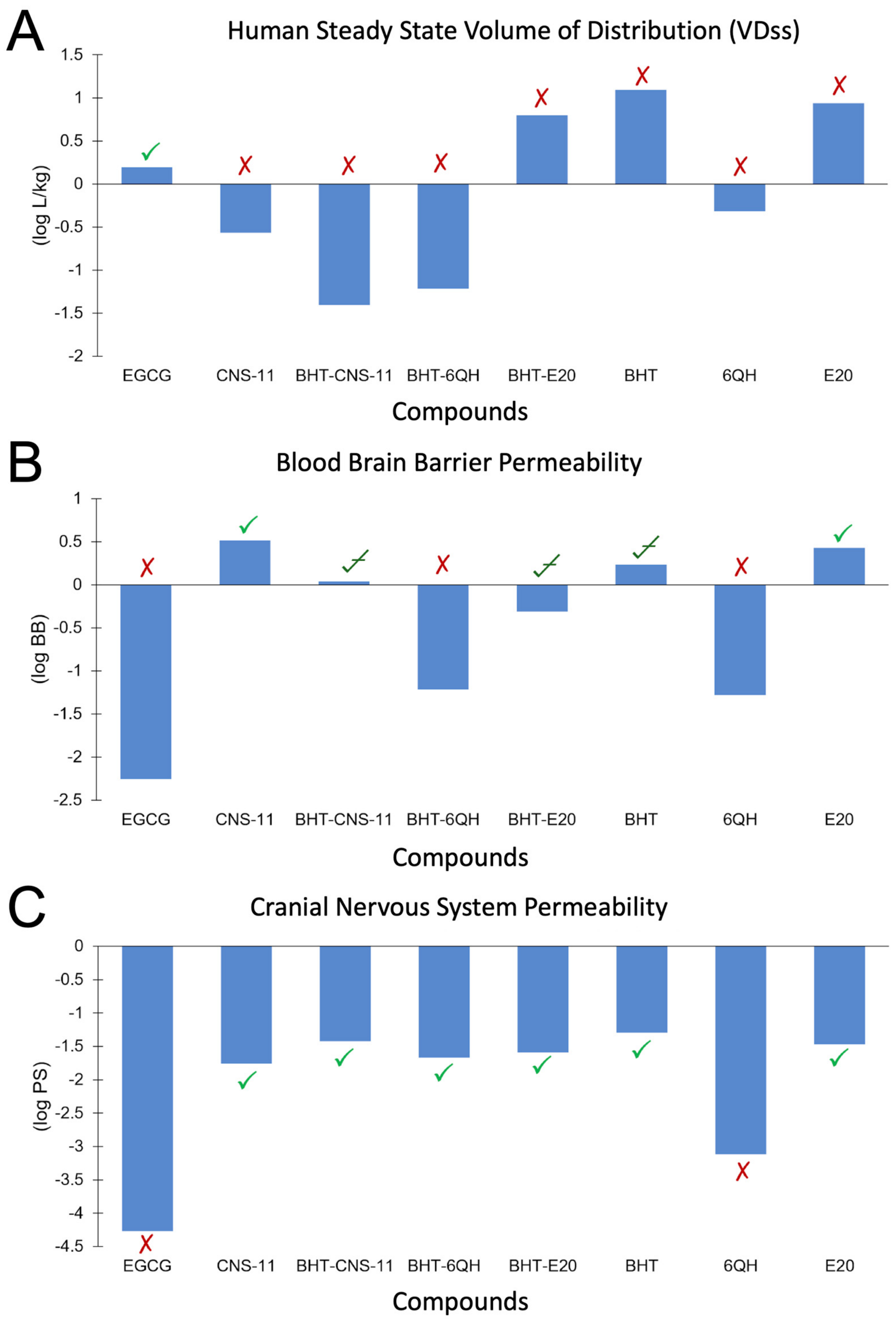

Figure S2. Distribution properties for compounds EGCG, CNS-11, BHT-CNS-11, BHT-6QH, BHT-E20, BHT, 6QH, and E20. The values of human steady-state volume of distribution or VDss (**A**), Blood Brain Barrier Permeability (**B**), and Cranial Nervous System Permeability (**C**) are shown with optimal values check-marked with green, moderate values half-check-marked in dark green, and suboptimal values crossed in red. See the main text for more details.

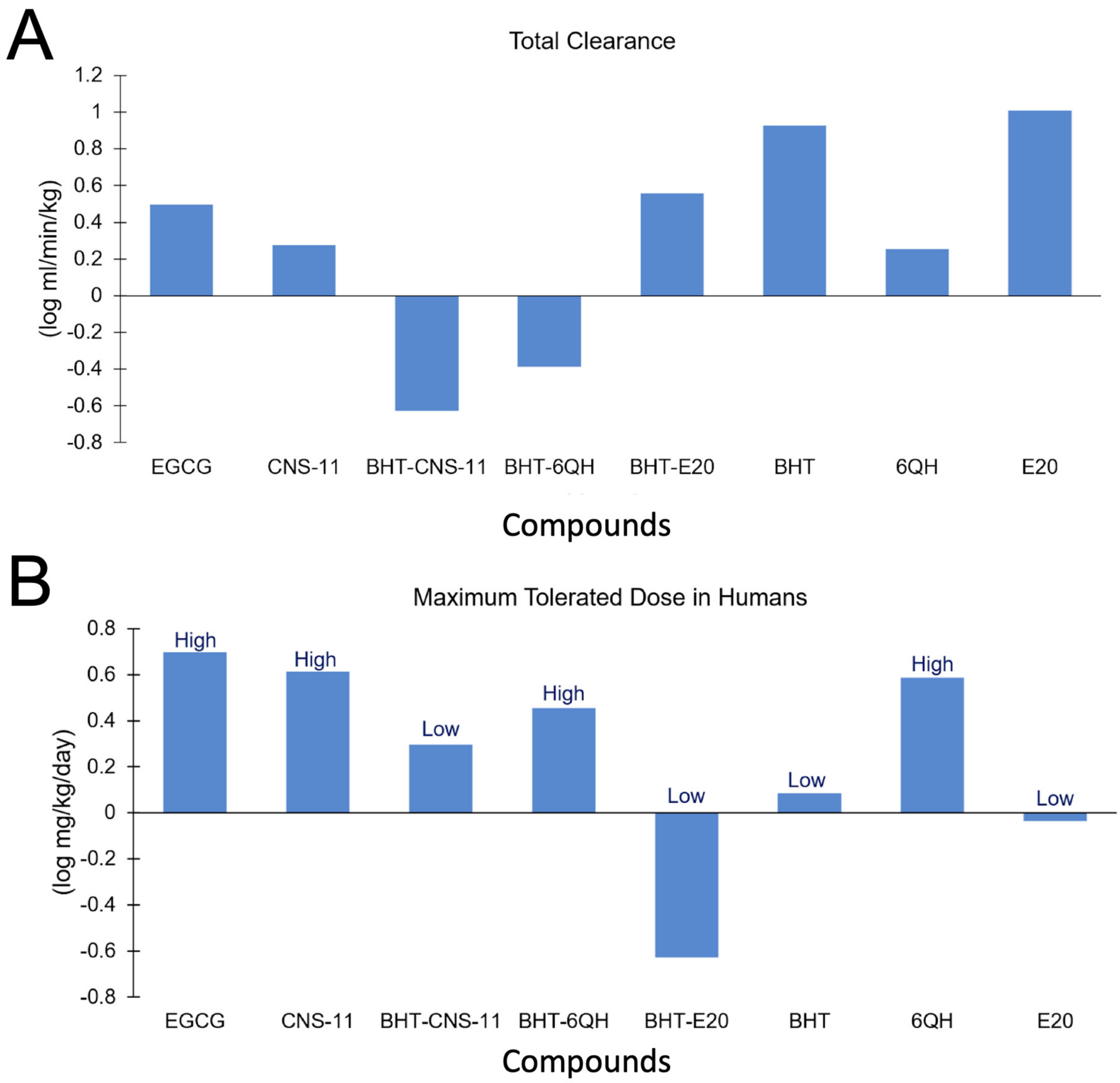

Figure S3. Excretion and toxicity properties of compounds EGCG, CNS-11, BHT-CNS-11, BHT-6QH, BHT-E20, BHT, 6QH, and E20. The total ability of the body to clear a compound from the plasma is displayed following a similar trend to human VDss (**A**). The highest recommended dose of a compound that does not cause unacceptable side effects is displayed (**B**).

Table S1. Compound Inhibition of Cytochrome P450 Isoforms.

Compound	CYP2D6[1] Inhibitor	CYP3A4[2] Inhibitor	CYP1A2[3] Inhibitor	CYP2C19[4] Inhibitor	CYP2C9[5] Inhibitor	CYP2D6[6] Substrate	CYP3A4[7] Substrate
EGCG	No	No	Yes	No	No	No	Yes
CNS-11	No	Yes	Yes	Yes	Yes	No	Yes
BHT-CNS-11	No	Yes	Yes	Yes	Yes	No	No
BHT-6QH	No	Yes	No	Yes	Yes	No	Yes
BHT-E20	No	Yes	No	No	No	No	Yes
BHT	No	Yes	Yes	No	No	No	No
6QH	No	Yes	Yes	No	No	No	No
E29	No	Yes	No	No	No	Yes	No

Yes/Inhibitor = Compound inhibits enzyme isoform; No/Inhibitor = Compound does not inhibit enzyme isoform; Yes/Substrate = The enzyme metabolizes the compound; No/Substrate = The enzyme metabolizes the compound.

[1,6] Cytochrome P450 family 2 subfamily D member 6 contributes to the metabolism of compounds and can be inhibited.

[2,7] Cytochrome P450 family 3 subfamily A member 4 is the most clinically relevant compound metabolizing enzyme in humans.

[3] Cytochrome P450 family 1 subfamily A member 2 is responsible for the metabolism of more than 100 clinically used drugs.

[4] Cytochrome P450 family 2 subfamily C member.

[5] Cytochrome P450 family 2 subfamily C member.

Table S2. Toxicity of Compounds.

Compound	AMES toxicity[1]	hERG I inhibitor[2]	hERG II inhibitor[3]	Hepatotoxicity[4]
EGCG	Yes	No	Yes	No
CNS-11	No	No	Yes	No
BHT-CNS-11	Yes	No	Yes	Yes
BHT-6QH	No	No	Yes	Yes
BHT-E20	No	No	Yes	Yes
BHT	No	No	No	No
6QH	No	No	No	Yes
E20	No	No	Yes	No

Yes/Toxicity = The compound is toxic; No/Toxicity = The compound is not toxic; Yes/Inhibitor = The compound inhibits; No/Inhibitor = The compound does not inhibit.

[1] The AMES test assesses the compound's mutagenic potential using bacteria where a positive test indicates that the compound reversed the bacteria mutation allowing for the bacteria to grow in the absence of histidine, acting as a carcinogenic.

[2,3] Human ether-a-go-go related gene (hERG) encodes the pore-forming subunits of rapidly activating delayed rectifier potassium channel, which is important for cardiac repolarization. A compound inhibiting the above process can lead to ventricular arrhythmias.

[4] A compound is classified as hepatotoxic if it has induced at least one side effect associated with the disruption of normal liver function.

Table S3. Molsoft-prediction of molecular properties and drug-likeness of compounds.

Compound	MW[1] (g/mol)	pKa Most Basic/Acidic[2]	H-bond Acceptor[1]	H-bond Donor[1]	Log P[3]	Log S[4]	PSA[5] (Å^2)	Drug-Likeness[5]
EGCG	458.08	< 0./8.07	11	8	1.44	-1.60	158.72	0.23
CNS-11	395.16	2.88/10.69	3	1	3.88	-4.24	48.73	-0.25
BHT-CNS-11	481.24	5.06/9.96	4	2	6.07	-5.48	65.60	-0.48
BHT-6QH	643.28	2.25/9.38	6	3	8.17	-5.92	89.32	0.65
BHT-E20	466.30	8.51/9.38	3	2	6.13	-5.51	43.18	0.93
BHT	220.18	<0./9.81	1	1	4.78	-4.60	15.72	-1.50
6QH	361.09	3.56/15.42	6	1	1.02	-2.56	76.19	0.54
E20	379.21	8.75/18.70	4	0	4.46	-4.31	32.39	1.56

The parameter in red represents the value outside the threshold or acceptable range.
[1] Thresholds of the Lipinski drug-like space: MW < 500, H-bond Acceptor ≤ 10, and H-bond donor ≤ 5.
[2] Thresholds: Highly acidic, < 2; moderately acidic, 2-6; near neutral, 6-8; slightly basic, 8-10; and highly basic, >10.
[2] Threshold of Log P (logarithm of the ratio between the compound's concentration in nonpolar solvent and water): 2 to 5
[3] Threshold of Log S (logarithm of water in mol/L at 25 °C: -4 to -6.
[4] Threshold of polar surface area (PSA): < 90 Å^2
[5] Acceptable range of drug-likeness: -2.0 to 2.0.

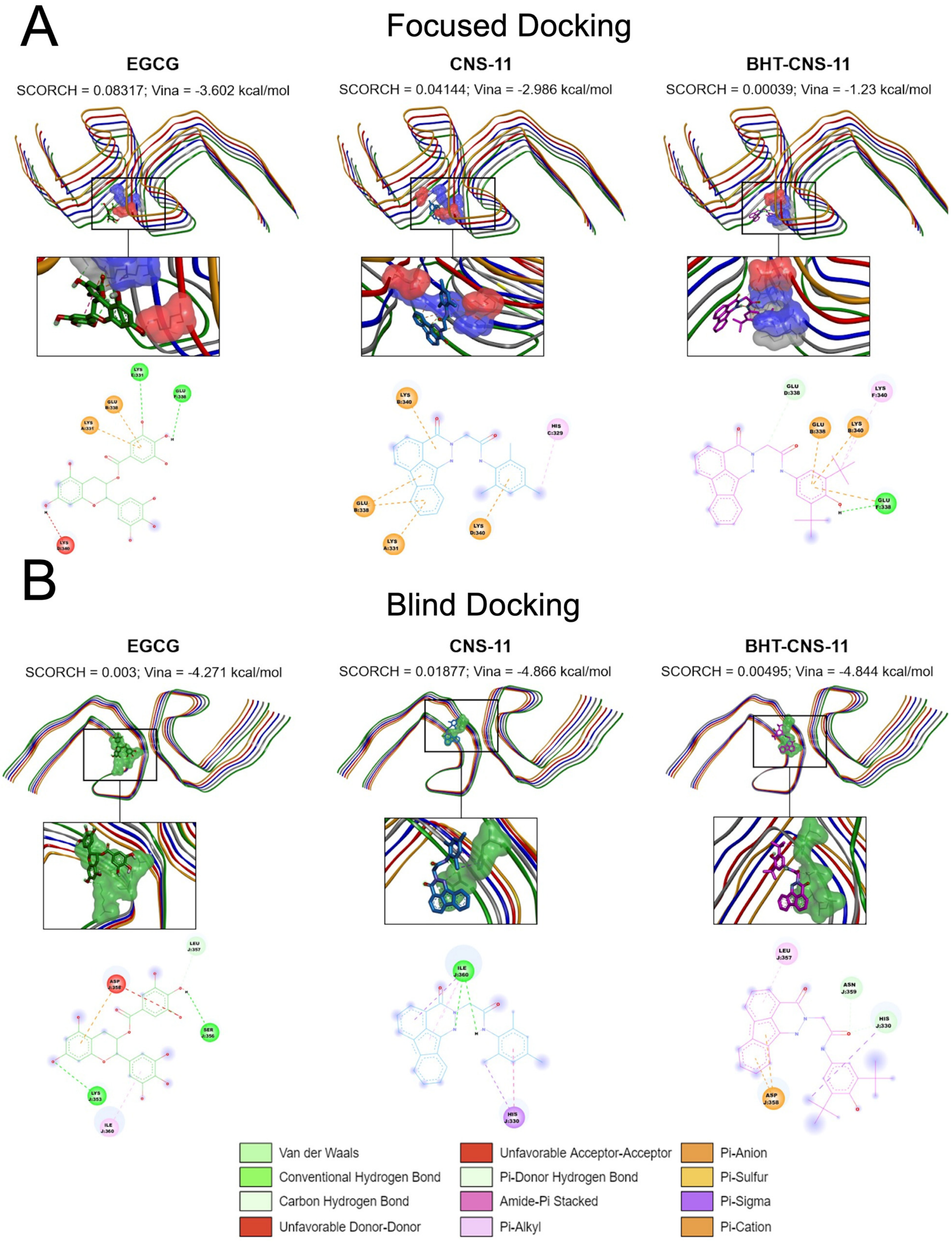

Figure S4. Docking of compounds EGCG (green), CNS-11 (blue), and BHT-CNS-11 (purple to tau-PHF-A presenting binding location, Autodock Vina binding affinity, SCORCH score, and 2D chemical interactions (last rows). The types of chemical interactions are color-coded, as shown at the bottom of the figure. Focused docking of compounds to tau-PHF-A with a fixed search box of 20Å x 16Å x 12Å (**A**) and blind docking without a pre-defined search box is shown (**B**). The interacting protein residues are displayed with the colored surfaces in each box.

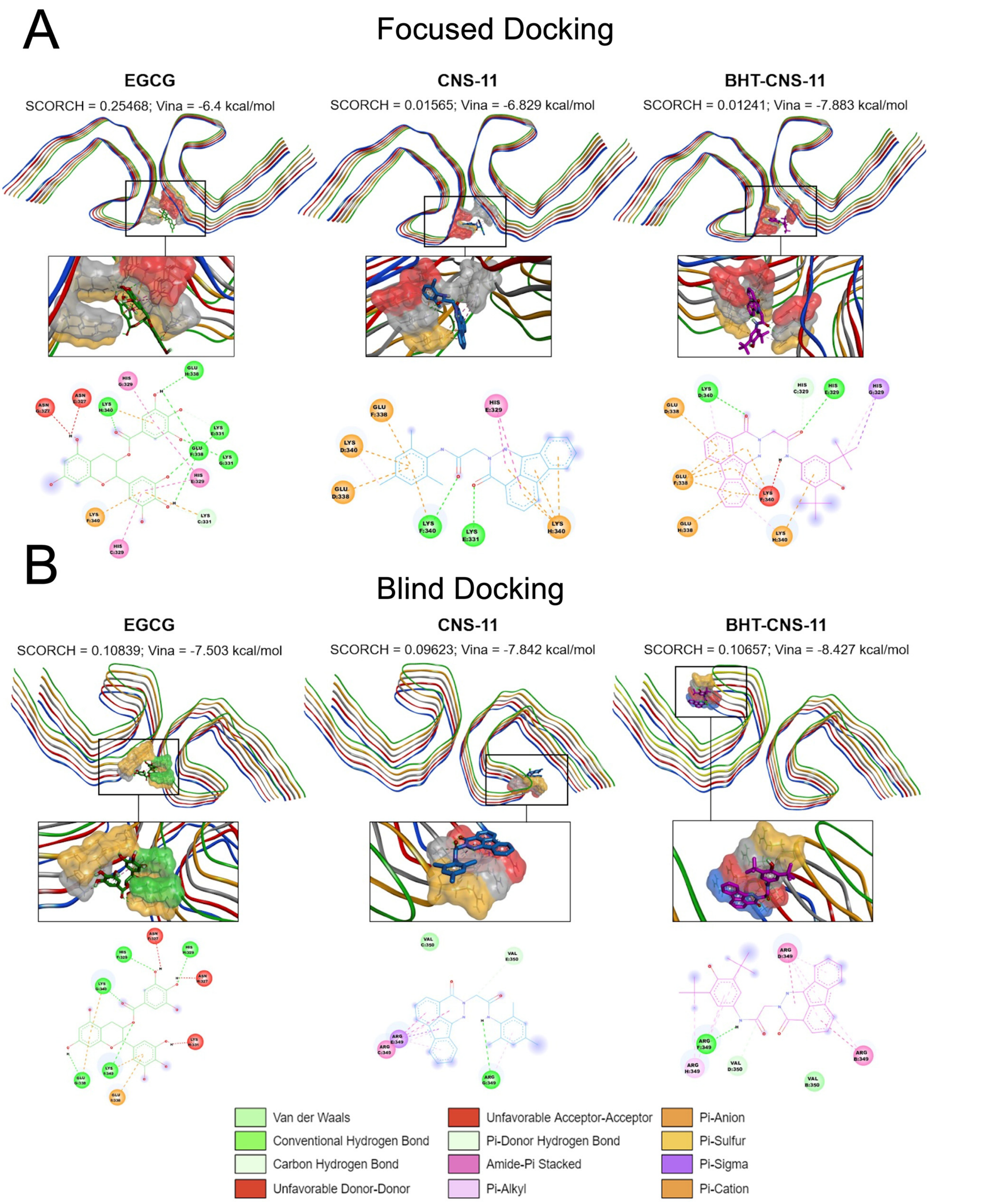

Figure S5. Docking of compounds EGCG (green), CNS-11 (blue), and BHT-CNS-11 (purple to tau-PHF-C presenting binding location, Autodock Vina binding affinity, SCORCH score, and 2D chemical interactions (last rows). The types of chemical interactions are color-coded, as shown at the bottom of the figure. Focused docking of compounds to tau-PHF-A with a fixed search box of 20Å x 16Å x 12Å (**A**) and blind docking without a pre-defined search box is shown (**B**). The interacting protein residues are displayed with the colored surfaces in each box.

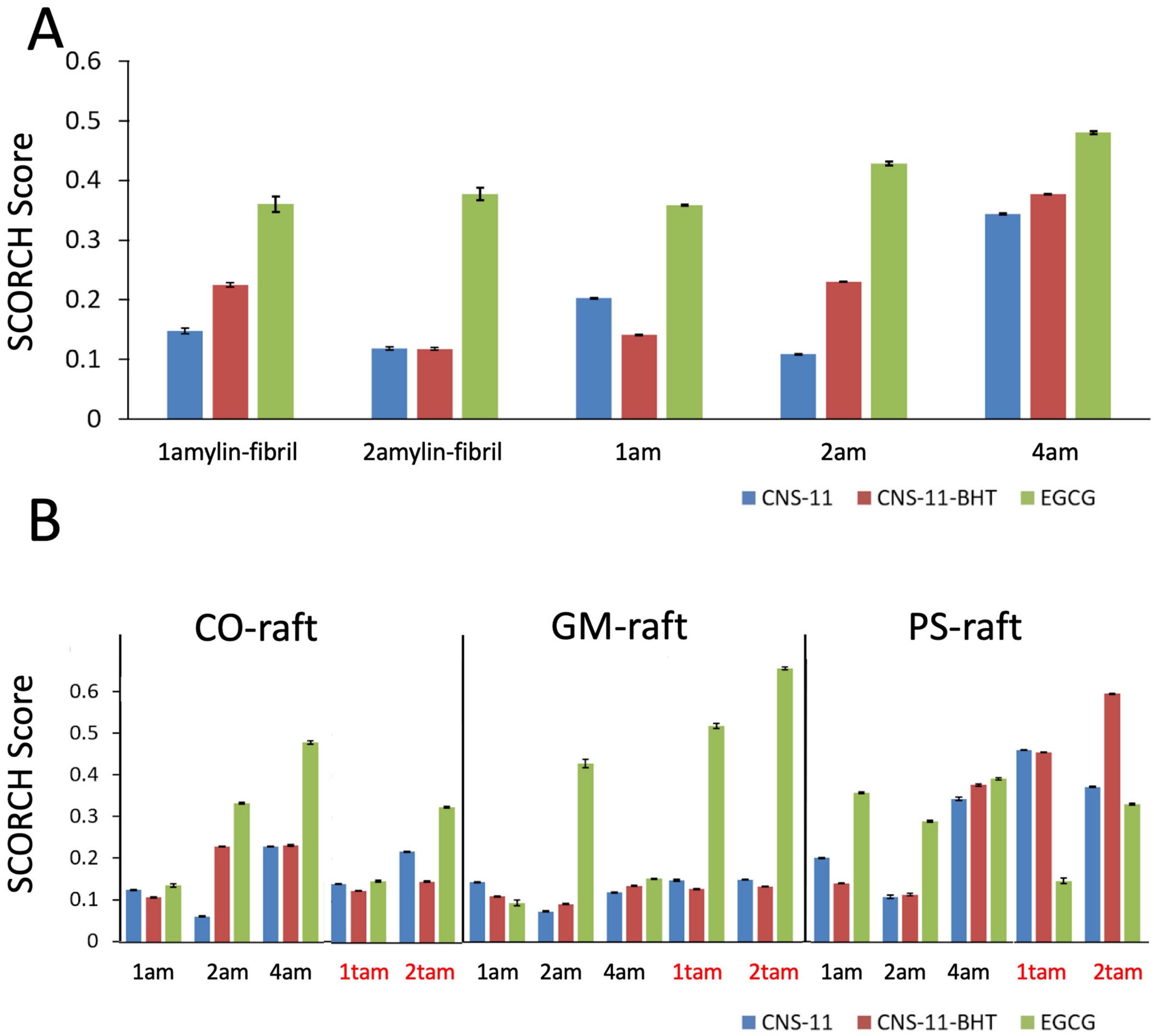

Figure S6. Single protofilament of Amylin Fibril (1amylin-fibril), symmetrically related protofilaments of Amylin (2amylin-fibril), amylin monomer (1am), amylin dimer (2am), and amylin tetramer (4am) are compared in terms of maximum SCORCH score for each of the top lead conformers of CNS-11, BHT-CNS-11, and EGCG (**A**). 1am, 2am, 4am, tau-amylin heterogeneous dimer (1tam), and tau-amylin heterogeneous tetramer (2tam) on different membrane-bound raft conditions (CO: control, GM: raft with ganglioside, PS: raft with phosphatidylserine) are compared in terms of maximum SCORCH score for each of the top lead conformers of CNS-11, BHT-CNS-11, and EGCG (**B**). Error bars were calculated by taking the standard error from the mean for all protein target replicates, molecular docking iterations (total of 4 iterations), and bound compound pose conformers. Out of this dataset, only the top score for each compound is displayed.

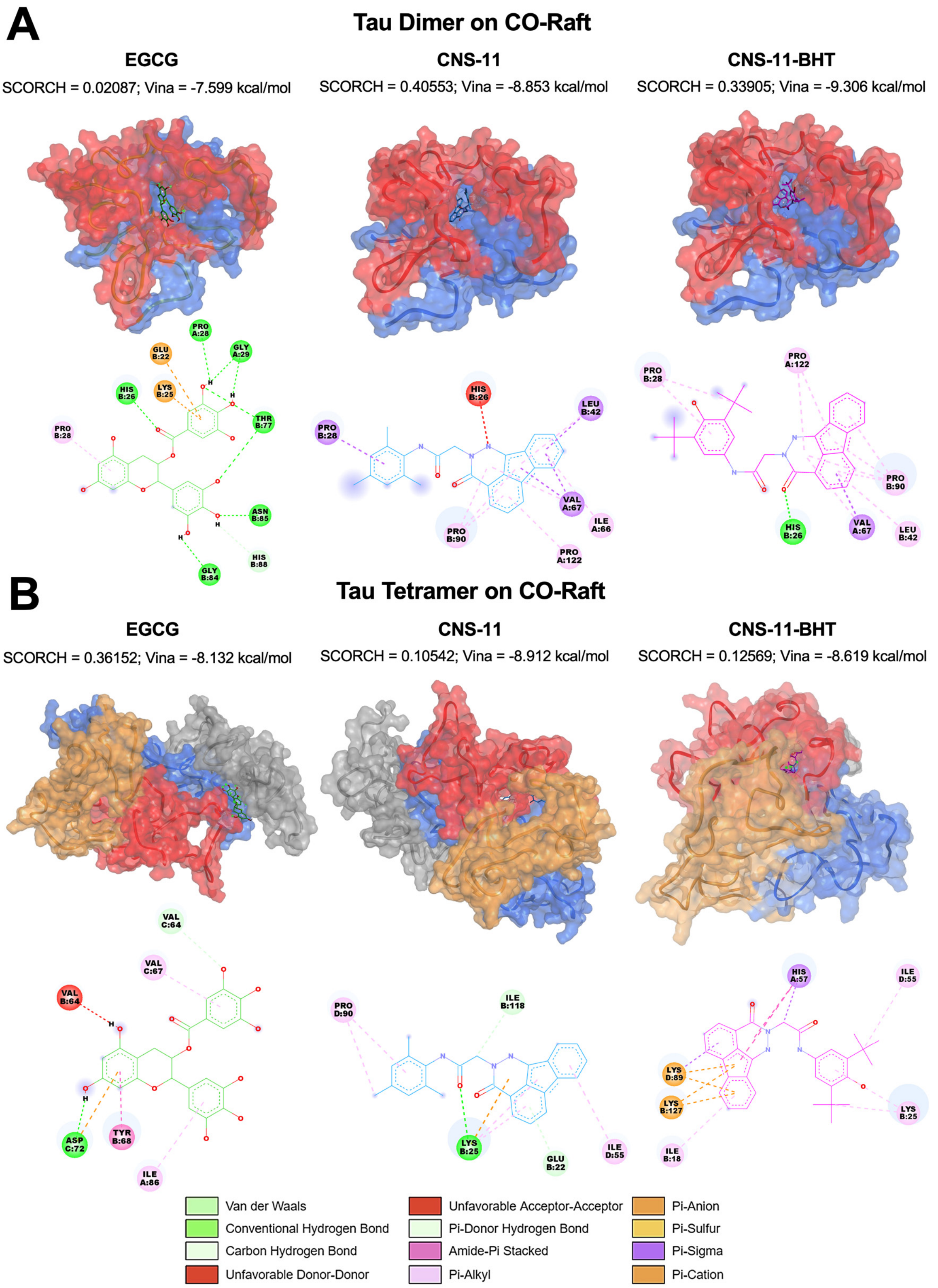

Figure S7. Docking of lead compounds to tau oligomers on CO-raft. The 3D structures and 2D chemical interactions of lead compounds, EGCG, CNS-11, and BHT-CNS-11, docked to tau dimer (**A**) and tetramer (**B**). The Vina and SCORCH scores are given. Tau chains are identified by colors. The types of chemical interactions are color-coded, as shown at the bottom of the figure.

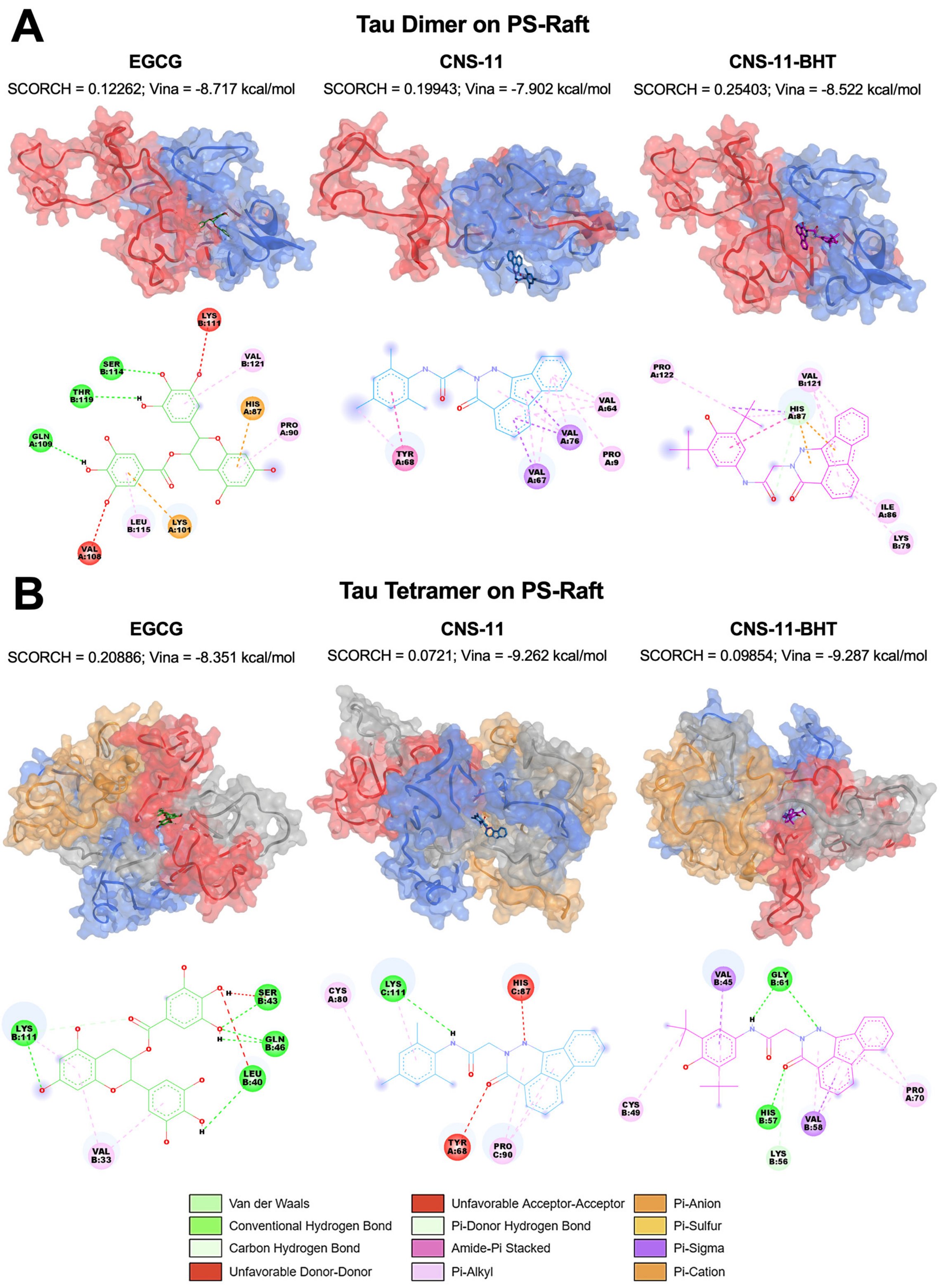

Figure S8. Docking of lead compounds to tau oligomers on PS-raft. The 3D structures and 2D chemical interactions of lead compounds, EGCG, CNS-11, and BHT-CNS-11, docked to tau dimer (**A**) and tetramer (**B**). The Vina and SCORCH scores are given. Tau chains are identified by colors. The types of chemical interactions are color-coded, as shown at the bottom of the figure.

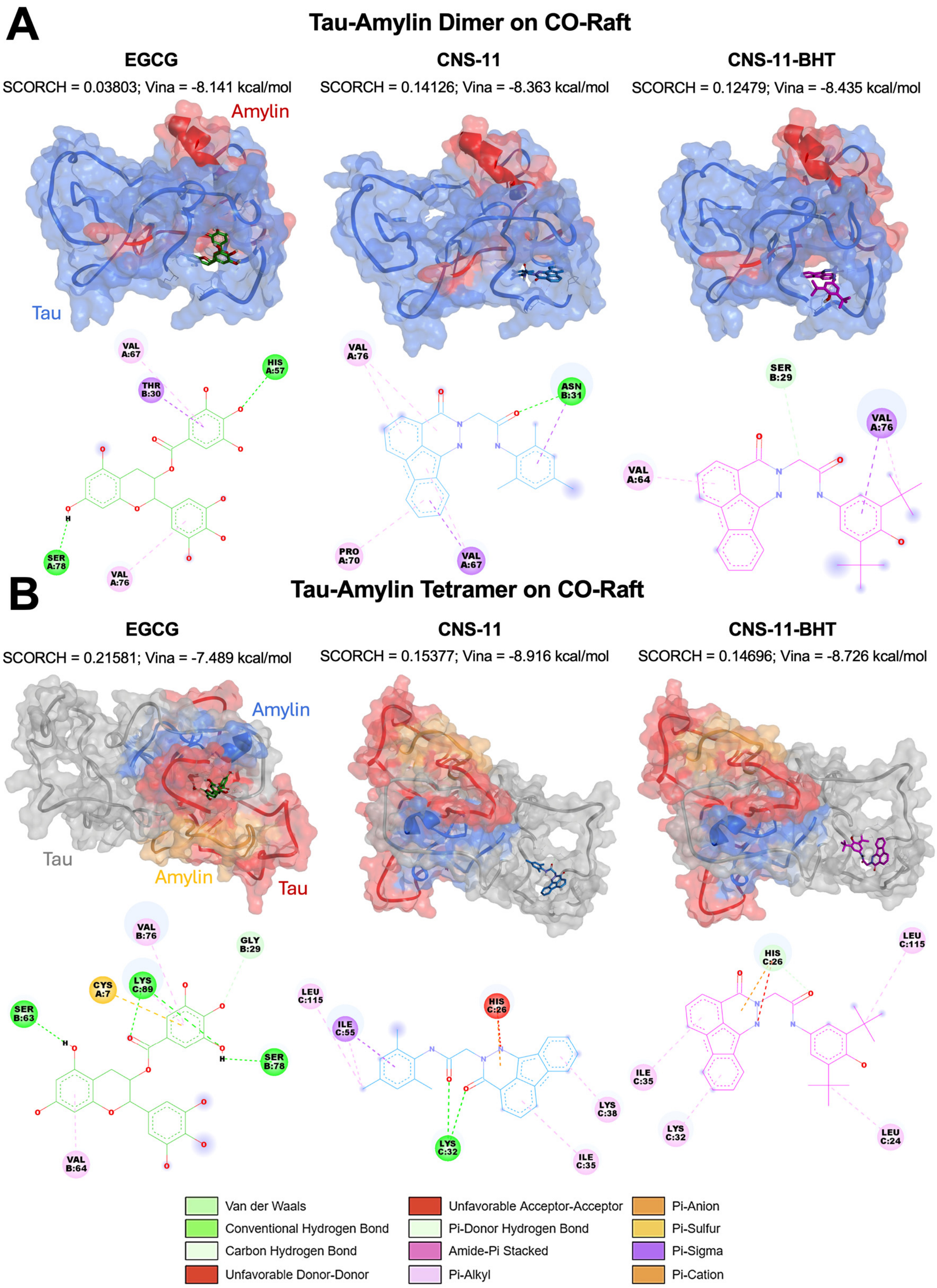

Figure S9. Docking of lead compounds to tau-amylin oligomers on CO-raft. The 3D structures and 2D chemical interactions of lead compounds, EGCG, CNS-11, and BHT-CNS-11, docked to tau dimer (**A**) and tetramer (**B**). The Vina and SCORCH scores are given. Tau and amylin chains are identified by colors. The types of chemical interactions are color-coded, as shown at the bottom of the figure.

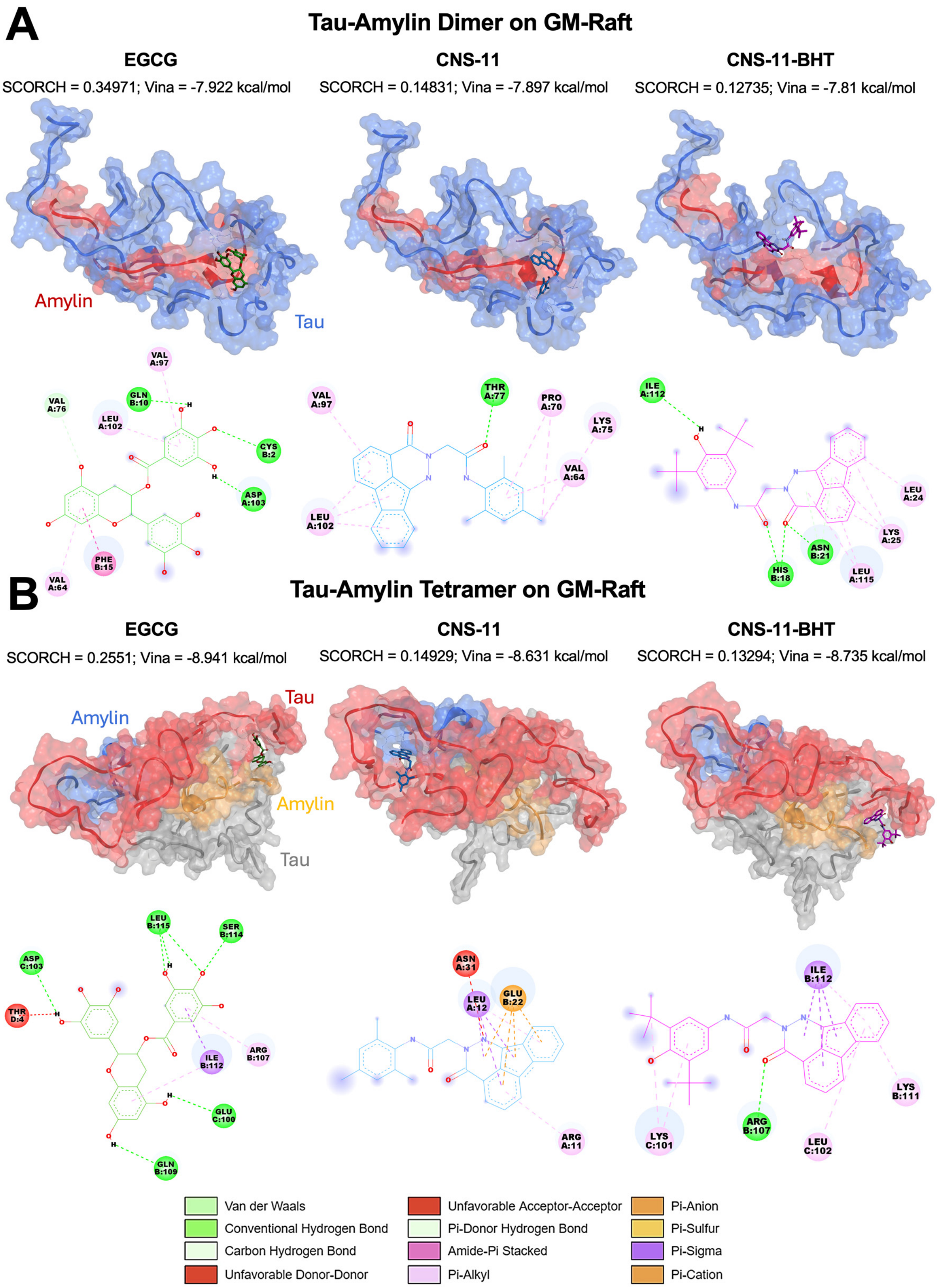

Figure S10. Docking of lead compounds to tau-amylin oligomers on GM-raft. The 3D structures and 2D chemical interactions of lead compounds, EGCG, CNS-11, and BHT-CNS-11, docked to tau dimer (**A**) and tetramer (**B**). The Vina and SCORCH scores are given. Tau and amylin chains are identified by colors. The types of chemical interactions are color-coded, as shown at the bottom of the figure.

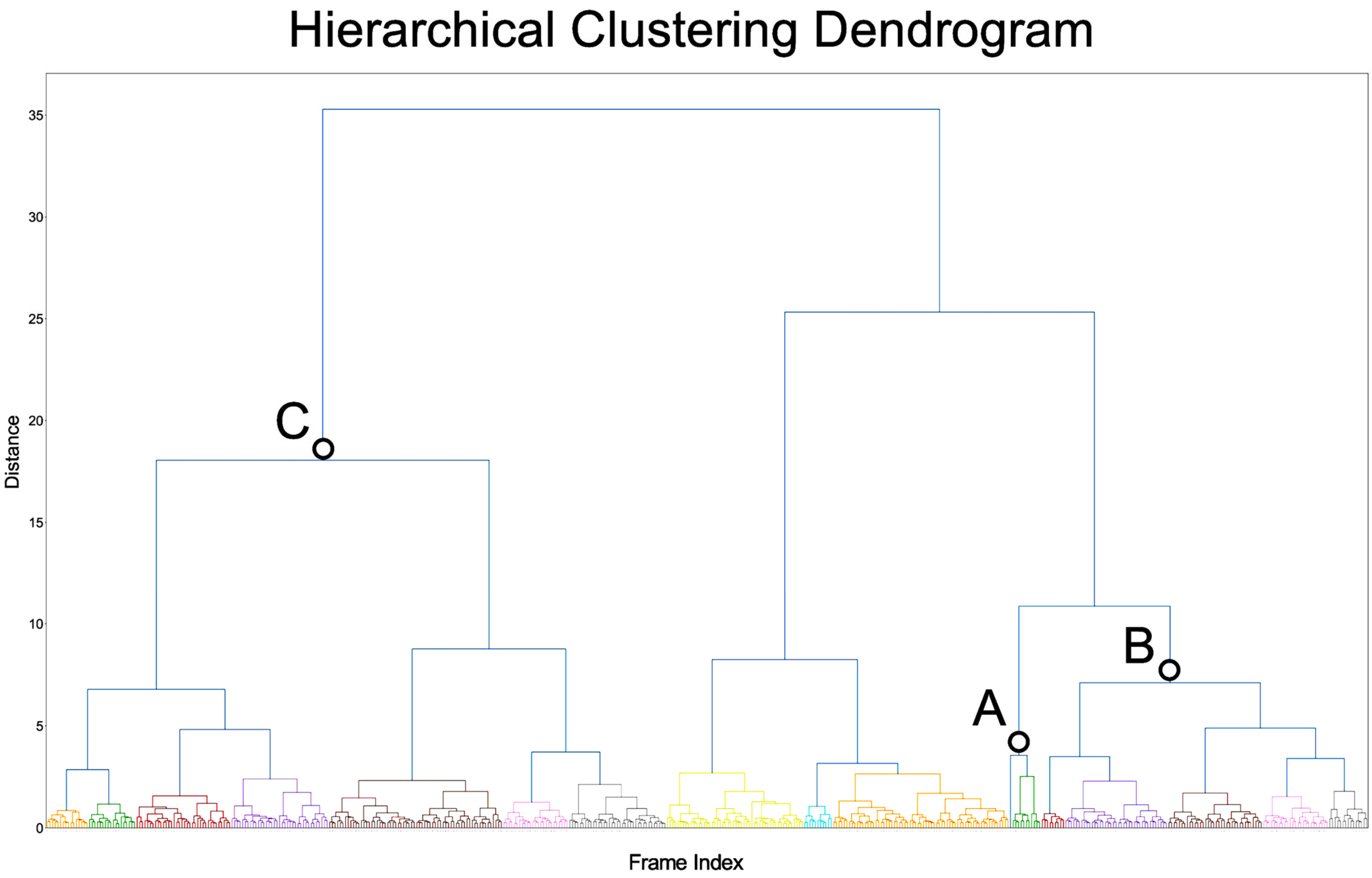

Figure S11. Hierarchical clustering dendrogram of time-dependent tau dimer structures. Letters A, B, and C indicate the nodes for each cluster from which each representative tau dimer frame was derived in Fig. S12.

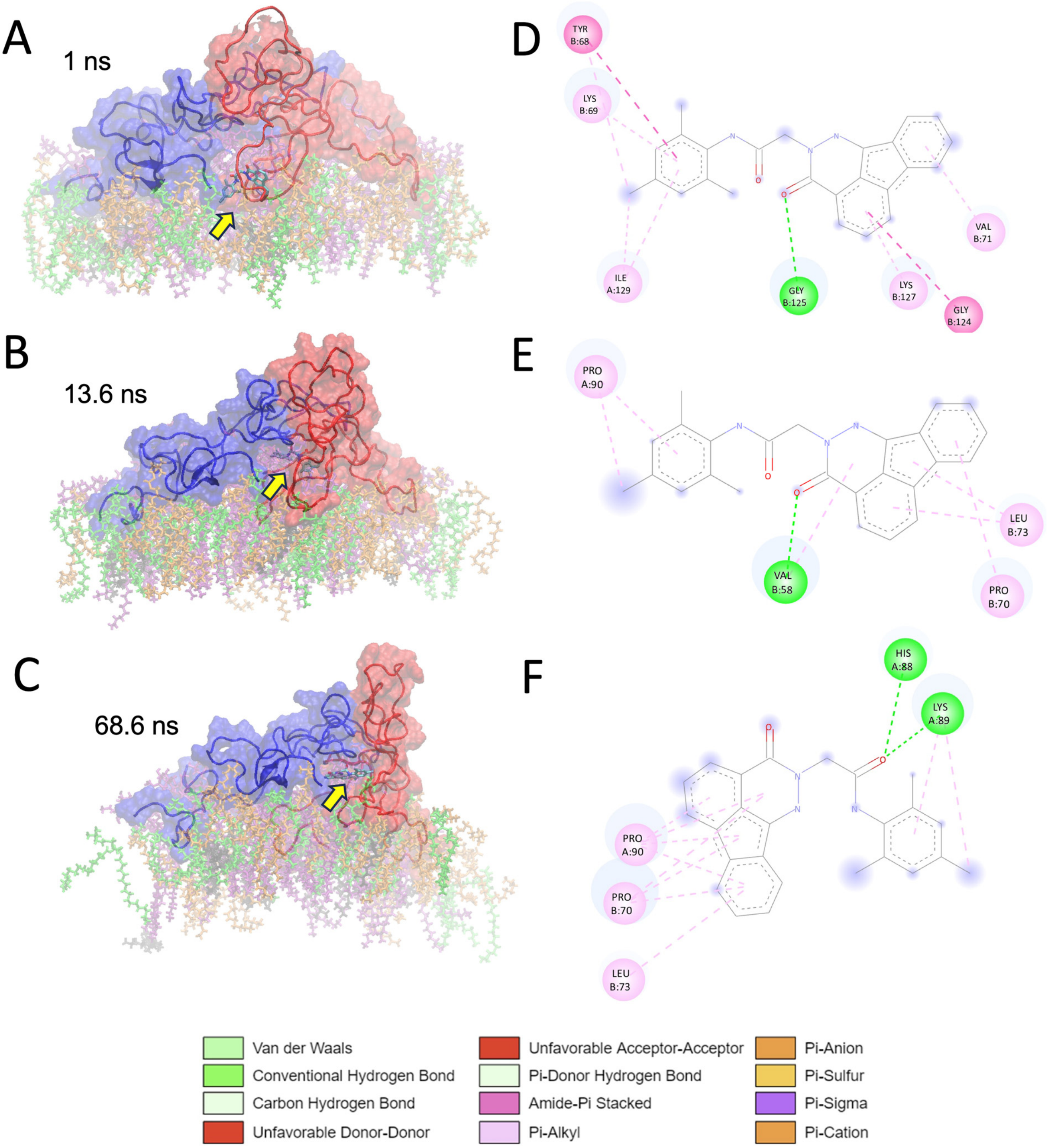

Figure S12. CNS-11 binding to tau-dimer on GM-raft at selected time frames. The locations of the bound CNS-11 molecule (yellow arrow) to tau dimer on GM-raft and the 2D chemical interactions at 1 ns (**A, D**), 13.6 ns (**B, E**), and 68.6 ns (**C, F**) are shown. The chains of the membrane-bound tau dimer are represented in colored ribbons and surfaces. The four interacting lipids of the GM-raft surrounding the tau dimer are color-coded with the dipalmitoyl phosphatidylcholine in green, dilinoleoyl phosphatidylcholine in orange, cholesterol in black, and GM1 in red. The types of chemical interactions are color-coded, as shown at the bottom of the figure.
